# A dual-drug sequential delivery hydrogel for programmatic microglia/macrophage polarization and function recovery in spinal cord injury

**DOI:** 10.1016/j.mtbio.2025.102365

**Published:** 2025-09-29

**Authors:** Ya Li, Yuyun Liang, Chaoyong He, Runxiang Yao, Ke Jian, Liyang Shi

**Affiliations:** a College of Biology, Hunan University, Changsha, 410082, China; b Institutes of Health Central Plain, Clinical Medical Center of Tissue Engineering and Regeneration, Xinxiang Medical University, Xinxiang, 453003, China

**Keywords:** Dual-drug, Sequential release, Microglia/macrophages, Programmable polarization, Spinal cord injury

## Abstract

Precisely modulating microglia/macrophage polarization to meet the dynamic needs of different stages after spinal cord injury (SCI) is a significant challenge due to the complexity of M1/M2 polarization processes. This study introduces a dual-drug sequential delivery hydrogel (DSDH) to modulate microglia/macrophage polarization through sequential-drug release. DSDH utilizes a hyaluronic acid-based hydrogel combined with fucoidan nanoparticles to deliver minocycline initially, suppressing M1 polarization during early acute inflammatory stage, and a biotin-streptavidin system to release interleukin-4 subsequently, promoting M2 polarization in late non-acute stage. In a rat model of SCI, DSDH programmatically modulated the inflammatory microenvironment, significantly reduced scar formation, and effectively enhanced neuron regeneration compared to dual-drug non-sequential delivery hydrogel (non-DSDH). Additionally, compared with non-DSDH, DSDH improved motor function, alleviated bladder dysfunction, and reduced lesion cavity. This study underscores the potential of temporal-controlled drug release for SCI immunotherapy, offering a promising strategy for addressing dynamic neuroinflammatory responses and improving functional recovery after SCI.

## Graphical abstract

**Figure:**
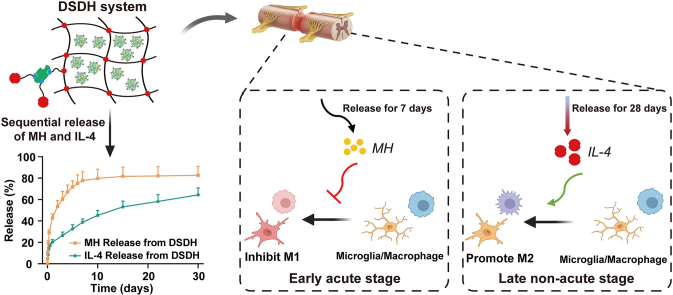

## 1 Introduction

Numerous physiological processes in the human body, such as tissue regeneration and immune responses, exhibit stringent spatiotemporal regulation [1,2]. For instance, tissue regeneration relies on the coordinated action of multiple small-molecule compounds, growth factors and cytokines, which are released in a temporally controlled manner to exert stage-specific biological effects [3,4]. Hence, tissue regeneration and immune regulation often require precisely timed delivery of multiple therapeutic agents, e.g., initial anti-inflammatory agents followed by pro-regenerative cytokines [5,6]. Programmable drug delivery systems represent a class of pharmaceutical platforms capable of precisely controlling the temporal sequence and spatial distribution of drug release to mimic physiological processes or meet therapeutic requirements [7,8]. The fundamental principle involves integrating drug release with biological cues or material properties to achieve dynamic regulation, enabling single-dose administration with multi-stage release [9]. These systems serve as precision delivery platforms capable of in situ programmable drug release to facilitate tissue regeneration [10,11] and disease treatment [7,12].

Spinal cord injury (SCI) generally results in sensory and motor dysfunction below the point of injury, leading to permanent loss of mobility and a profound impact on the patient's quality of life [13,14]. The pathological processes of SCI consist of primary injury and secondary injury, with inflammation playing a key role in the secondary injury and exacerbating further damage [15,16]. The primary immune cells involved in the inflammatory response during SCI are microglia (resident in the nervous system) and macrophages (infiltrating from the vascular endothelium). These cells help regulate the inflammatory environment by secreting various cytokines. In the injured area, microglia/macrophages can be polarized into the “classically activated” M1 phenotype in response to pro-inflammatory factors like tumor necrosis factor (TNF)-α. M1 microglia/macrophages release more pro-inflammatory cytokines, worsening the injury and promoting scar formation [17,18]. On the other hand, microglia/macrophages can also shift to the “alternatively activated” M2 phenotype when exposed to such factors as interleukin 4 (IL-4). M2 microglia/macrophages secrete anti-inflammatory cytokines and neurotrophic factors, which can help support spinal cord functional remodeling. After injury, resting microglia/macrophages (M0) quickly polarize into the M1 phenotype, which predominates in the first week after injury. M2 microglia/macrophages emerge later, typically after one week, in smaller numbers [19]. Because the M1 and M2 polarization responses occur at different times after SCI, programmable drug delivery systems are required to address the spatiotemporal dynamics and complexity of microglial/macrophage polarization.

Minocycline hydrochloride (MH), a tetracycline antibiotic, effectively inhibits the polarization of microglia/macrophages toward the M1 phenotype at the SCI site, without affecting M2 cells. This drug exhibits strong anti-inflammatory, antioxidant, anti-apoptotic, and neuroprotective properties, significantly improving neural function [20]. IL-4, on the other hand, promotes the polarization of microglia/macrophages toward the M2 phenotype by activating signaling pathways such as PI3K/Akt and JAK1/STAT6. When delivered through intramedullary, intraperitoneal, or local administration, IL-4 reduces the accumulation of inflammatory factors at the injury site, enhances nerve regeneration, and partially improves motor function [21]. Although some progress has been made in using minocycline or IL-4 alone to regulate microglia/macrophage polarization, these approaches do not yield a synergistic effect for SCI treatment [[22], [23], [24]]. Hydrogels, recognized as a novel generation of drug delivery systems, are widely utilized in drug platforms due to their exceptional biocompatibility and modifiability. Recent developments in sequential drug delivery hydrogel, which combine the advantages of hydrogels and sequential release systems, provide a promising solution for addressing the ever-changing SCI microenvironment and enabling precise, controlled drug release [25]. For instance, Dai et al. employed a collagen-fibrin (Col-FB) fibrous hydrogel to achieve spatiotemporal delivery of stromal cell-derived factor-1α (SDF1α) and paclitaxel (PTX), where SDF1α was released first to recruit endogenous neural stem/progenitor cells (NSPCs) in SCI area, followed by PTX to enhance the neuronal differentiation rate of the recruited NSPCs [26]. Moreover, an “Inner-Outer” structure of fiber-hydrogel scaffold was capable of releasing anti-CD80 monoclonal antibody and Metformin sequentially, enabling them to perform their protective functions at distinct SCI stages [27]. Hyaluronic acid (HA) is a highly hydrophilic glycosaminoglycan composed of repeating disaccharide units (D-glucuronic acid and N-acetylglucosamine). As a key component of the extracellular matrix, HA is ubiquitously distributed in connective tissues, epithelial tissues, and neural tissues, demonstrating exceptional biocompatibility [28]. Furthermore, numerous studies have demonstrated that HA-based hydrogels exhibit remarkable neuroprotective properties and show tremendous potential for SCI repair and regeneration [29]. Therefore, we hypothesize that codelivery of MH and IL-4 using HA-based hydrogel for dual-drug sequential release can effectively address the dynamic changes in microglia/macrophage polarization at the lesion site.

In this study, we introduced a novel HA-based dual-drug sequential delivery hydrogel (DSDH) for programmatical microglia/macrophage polarization through the co-delivery of MH and IL-4. The DSDH incorporated MH-loaded fucoidan (Fuc) nanoparticles (MH@Fuc NPs), formed through electrostatic interactions between MH and Fuc, along with HA derivatives that provided the hydrogel backbone. A biotin-streptavidin system further enhanced the delivery of IL-4, enabling controlled, sustained release of the protein drug. In vitro studies showed that DSDH released MH over a period of seven days and IL-4 over thirty days. In SCI rat models, DSDH effectively suppressed M1 polarization within the first week, followed by promotion of M2 polarization at the four-week mark, addressing the changing polarization dynamics during SCI. Notably, DSDH significantly improved motor function recovery, alleviated bladder dysfunction, and reduced lesion cavities compared to non-programmed systems. This dual-drug, sequential release approach provides a promising strategy for programmatically regulating microglia/macrophage polarization in SCI therapy.

## 2 Results

### 2.1 Design, preparation and characterization of MH delivery system

**Fig. 1:**
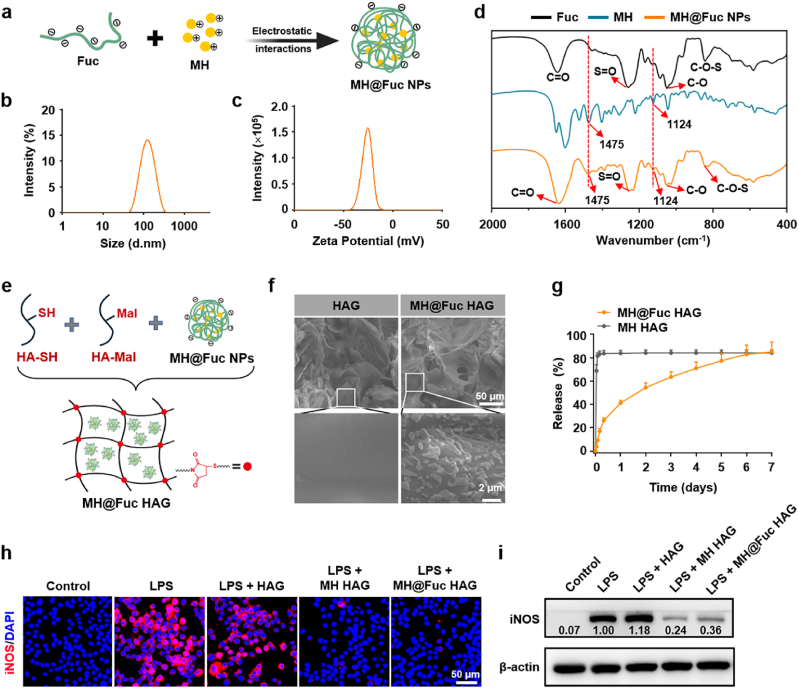
**Design, preparation and characterization of MH delivery system.** (a) Schematic for the fabrication of MH-loaded fucoidan (Fuc) nanoparticles (MH@Fuc NPs). (b) Hydrodynamic size of MH@Fuc NPs using DLS measurement. (c) Zeta potential of MH@Fuc NPs. (d) Infrared spectra of Fuc, MH, and MH@Fuc NPs. (e) Schematic illustration of the fabrication process for MH@Fuc NPs loaded hyaluronic acid (HA) hydrogel (i.e., MH@Fuc HAG). (f) Microscopic morphology of freezing-dried drug-free HA hydrogel (HAG) and MH@Fuc HAG. (g) MH release curves of MH@Fuc HAG and MH HAG (where MH was directly loaded into HAG without MH@Fuc NPs) (n = 3). (h) Immunofluorescence staining (red, iNOS; blue, cell nuclear) and (i) protein expression level of iNOS in BV2 cells treated by the drug-releasing solution of HAG, MH HAG and MH@Fuc HAG for 24 h in the presence of LPS. Data are presented as mean ± SD. (For interpretation of the references to color in this figure legend, the reader is referred to the Web version of this article.)

To engineer the MH delivery system, MH@Fuc NPs were first constructed by simply mixing Fuc and MH through electrostatic interactions (Fig. 1a and Fig. S1). When the MH solution was mixed with the Fuc solution under ultrasonic conditions, the mixture gradually became turbid. After centrifugation, a yellow precipitate formed at the bottom, indicating that MH participated in the formation of nanoparticles (Fig. S1). To investigate the role of electrostatic interactions in the assembly of MH@Fuc NPs, the nanoparticles were prepared under weakly acidic (pH = 6.0), neutral (pH = 7.0), and weakly alkaline conditions (pH = 8.0), as these pH levels can interfere with electrostatic interactions and coordination bonding (Fig. S2a). Particles formed only under neutral and weakly alkaline conditions, suggesting that electrostatic interactions play a critical role in the assembly of MH@Fuc NPs. Since metal ions were not used in the preparation process, coordination bonding was excluded as a mechanism. Given that MH carries a positive charge and Fuc carries a negative charge, it was concluded that MH@Fuc NPs were formed primarily through electrostatic interactions.

Next, the preparation conditions for MH@Fuc NPs were optimized by adjusting the mass ratio of MH to Fuc. As the amount of Fuc increased relative to a fixed amount of MH, the supernatant gradually became lighter in color, and the amount of yellow precipitate at the bottom increased, indicating greater MH participation in nanoparticle formation (Fig. S2b). The physicochemical properties of nanoparticles prepared at different ratios were then measured. As the Fuc ratio increased, the polydispersity index (PDI) of the nanoparticles initially decreased and then slightly increased, while the encapsulation efficiency (EE) of MH gradually increased and eventually stabilized. At a mass ratio of MH to Fuc of 1:15, the PDI was the lowest, indicating the best uniformity of nanoparticles (Table S1). Additionally, the EE of MH reached its highest value of 95.76 % at this ratio. Therefore, a mass ratio of 1:15 was selected for the preparation of MH@Fuc NPs to ensure optimal uniformity and high encapsulation efficiency.

Dynamic light scattering (DLS) analysis revealed that the MH@Fuc NPs were negatively charged, with a hydrodynamic diameter of 121.3 ± 0.1 nm (Fig. 1b and c and Table S1). The loading content (LC) of MH in the MH@Fuc NPs was approximately 60 %. Fourier transform infrared spectroscopy (FT-IR) analysis confirmed that the MH@Fuc NPs retained the spectral features of MH (Fig. 1d). In the FT-IR spectra of Fuc, peaks at 1643, 1259, 1086, and 843 cm^−1^ were assigned to C=O, S=O, C-O stretching vibrations, and C-O-S bending vibrations, respectively [17,18]. In the FT-IR spectra of MH@Fuc NPs, the characteristic peaks of Fuc remained unchanged, and new peaks at 1124 and 1475 cm^−1^ appeared in the FT-IR spectra of MH@Fuc NPs, confirming the successful encapsulation of MH [30,31].

Subsequently, thiolated HA (HA-SH) and maleimide modified HA (HA-Mal) were prepared via a condensation reaction and verified using ^1^H NMR (Figs. S3 and S4). We utilized thiol-maleimide approach to fabricate HA hydrogel (HAG). When HA solution was mixed with HA-SH or HA-Mal solutions, the storage modulus (G′) remained lower than the loss modulus (G″), indicating that no hydrogel formed (Fig. S5a and S5b). However, when HA-SH and HA-Mal solutions were mixed, G′ exceeded G″, confirming hydrogel formation (Fig. S5c). This demonstrated that both thiol and maleimide groups are essential for HAG formation. The mechanical strength of spinal cord tissue typically ranges between 100 and 3000 Pa [32], and the G′ of HAG fell within 100–1000 Pa, matching the modulus of spinal cord tissue without causing secondary injury and providing suitable mechanical support for tissue regeneration. MH@Fuc NPs were then loaded into the blank HAG to create MH@Fuc HAG (Fig. 1e). The G′ of MH@Fuc HAG remained higher than G″, indicating that the loading of MH@Fuc NPs did not interfere with the crosslinking of thiol and maleimide groups to form the gel network (Fig. S6). Scanning electron microscopy (SEM) revealed highly interconnected porous microstructures in both HAG and MH@Fuc HAG, which are suitable for controlled drug release and substance exchange, such as exudates (Fig. 1f). SEM also showed particles on the polymer sheets of MH@Fuc HAG, likely corresponding to the loaded MH@Fuc NPs (Fig. 1f).

To evaluate drug release behavior, in vitro monitoring of MH release from both MH HAG (where MH was directly loaded into HAG without MH@Fuc NPs) and MH@Fuc HAG was conducted (Fig. 1g). Similar to previously reported hydrogels for MH release [24,33], MH HAG released MH within 24 h, which is insufficient for the required one-week release period needed to inhibit M1 polarization of microglia/macrophages. In contrast, MH@Fuc HAG achieved a cumulative MH release of approximately 41 % by the 24th hour, with the release rate gradually increasing over time. By the 7th day, the cumulative release reached approximately 85 %. These results suggest that the self-assembly of Fuc and MH created strong interactions, preventing premature release.

To demonstrate the anti-inflammatory effects of MH@Fuc HAG, a lipopolysaccharide (LPS)-induced inflammation model was established using the BV2 cell line. Immunofluorescence staining and Western blotting of the pro-inflammatory marker inducible nitric oxide synthase (iNOS) were performed to evaluate microglia polarization after different treatments under LPS conditions. Compared to the LPS group, treatment with HAG alone did not prevent the polarization of LPS-induced BV2 cells into a pro-inflammatory phenotype (Fig. 1h and Fig. S7). However, MH HAG treatment showed the ability to regulate microglia polarization. Notably, a significant reduction in iNOS-positive BV2 cells was observed in the MH@Fuc HAG group. The protein expression of iNOS was elevated in the LPS group but significantly reduced in the MH@Fuc HAG group, confirming the anti-inflammatory polarization effect of MH@Fuc HAG (Fig. 1i). These results can be attributed to the anti-inflammatory effects of the released MH.

### 2.2 Design, preparation and characterization of protein drug delivery platform

**Fig. 2:**
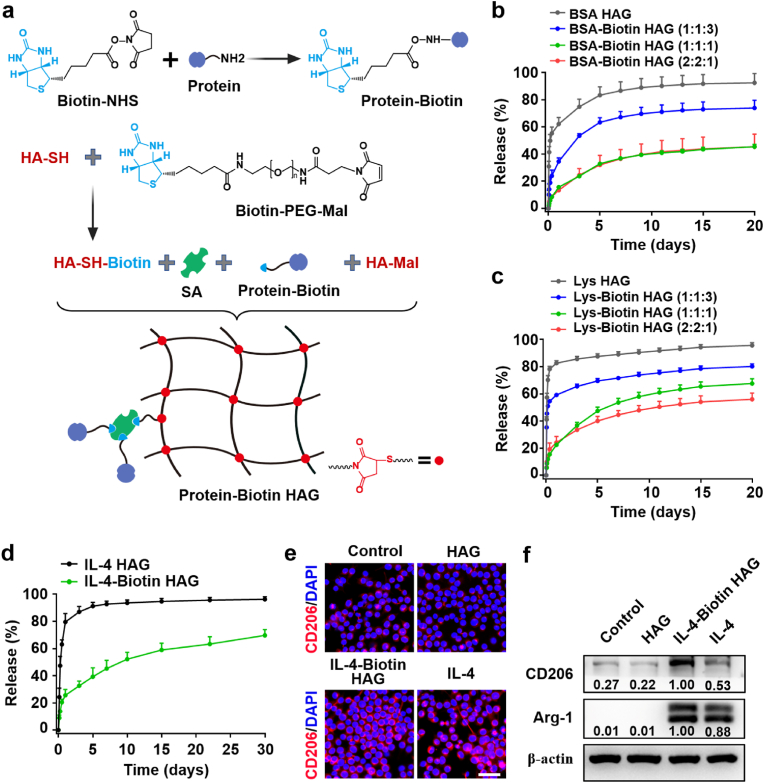
**Design, preparation and characterization of protein drug delivery platform.** (a) Schematic for fabrication of Protein-Biotin and Protein-Biotin HAG. BSA (b) and Lys (c) release curves (n = 5). (d) IL-4 release curves (n = 4). (e) Immunofluorescence staining of CD206 (red, CD206; blue, cell nuclear) and (f) protein expression level of CD206 and Arg-1 in BV2 cells treated by the HAG, IL-4-Biotin HAG and IL-4 for 24 h. Data are presented as mean ± SD. (For interpretation of the references to color in this figure legend, the reader is referred to the Web version of this article.)

To develop the protein drug delivery platform, HA derivatives were used as the backbone molecules. These were combined with the biotin-streptavidin system to create a hydrogel delivery system for protein drugs (Fig. 2a). Specifically, Biotin-NHS was first grafted onto the amino groups of the protein through a reaction between the N-hydroxysuccinimide (NHS) group and the amino group, forming Protein-Biotin. Simultaneously, Biotin-PEG-Mal was grafted onto the HA-SH molecular chain through a Michael addition reaction between the thiol and maleimide groups, resulting in HA-SH-Biotin. Next, Protein-Biotin was linked to HA-SH-Biotin using streptavidin (SA) to form HA-SH-Biotin-SA/Protein-Biotin. Finally, the hydrogel loaded with protein (Protein-Biotin HAG) was obtained through the Michael addition reaction between the thiol group and maleimide group of HA-SH-Biotin-SA/Protein-Biotin and HA-Mal.

To assess the sustained release of protein drugs from the platform, two model proteins were tested: bovine serum albumin (BSA) (MW 66.5 kDa, pI = 4.7) and lysozyme (Lys) (MW 14.4 kDa, pI = 11), which have negative and positive charges, respectively (Fig. 2b and c). The influence of the biotin-streptavidin system on the release profile was evaluated by varying the molar ratios of Biotin-PEG-Mal, SA, and Biotin-NHS (1:1:3, 1:1:1, and 2:2:1). Non-biotinylated proteins were also used to prepare control hydrogels, referred to as BSA HAG and Lys HAG. The release of proteins from BSA HAG and Lys HAG was measured, revealing that 62.10 % of BSA and 82.64 % of Lys were released within 1 day. However, when the molar ratio of Biotin-PEG-Mal, SA, and Biotin-NHS was 1:1:3, only 34.54 % of BSA and 59.22 % of Lys were released within 1 day. By 20 days, BSA-Biotin HAG and Lys-Biotin HAG released 73.91 % of BSA and 80.19 % of Lys, respectively. These results suggest that the biotin-streptavidin affinity system effectively slowed down protein release. The release rates of BSA and Lys decreased as the SA ratio increased. When the ratio of Biotin-PEG-Mal, SA, and Biotin-NHS was 2:2:1, the cumulative release rates of BSA and Lys no longer significantly decreased. Based on these findings, the ratio of Biotin-PEG-Mal, SA, and Biotin-NHS was set to 2:2:1 for subsequent studies. These in vitro release studies provide strong evidence that the protein drug delivery platform (Protein-Biotin HAG) is capable of controlled release for both positively and negatively charged model proteins.

Next, we tested whether the platform could achieve controlled and prolonged release of IL-4. To this end, IL-4 was encapsulated in the protein drug delivery platform using the 2:2:1 molar ratio of Biotin-PEG-Mal, SA, and Biotin-NHS, resulting in IL-4-Biotin HAG. Non-biotinylated IL-4 was used to prepare a control hydrogel (referred to as IL-4 HAG). The release profiles of IL-4 from IL-4 HAG and IL-4-Biotin HAG were analyzed by ELISA. IL-4 HAG exhibited a burst release, with 79.54 % of IL-4 released within 1 day (Fig. 2d). In contrast, IL-4-Biotin HAG released only 25.69 % of IL-4 on 1 day. IL-4 HAG released 92.67 % of IL-4 by the 7 days, and the cumulative release plateaued thereafter. Meanwhile, IL-4-Biotin HAG released 45.29 % and 69.68 % of IL-4 by the 7 and 30 days, respectively. These results indicate that the IL-4-Biotin HAG system, with its biotin-avidin affinity, effectively anchored IL-4 within the hydrogel, providing a stable platform for controlled release. This is consistent with the release profiles observed for the model proteins.

Furthermore, we evaluated the effect of IL-4-Biotin HAG on the polarization of BV2 cells towards the M2 phenotype. After 24 h of treatment with IL-4-Biotin HAG, immunofluorescence staining revealed a significantly higher number of CD206-positive BV2 cells compared to the Control and HAG groups, similar to the IL-4 group. This suggests that IL-4-Biotin HAG significantly promotes BV2 cells polarization towards the M2 phenotype (Fig. 2e and Fig. S8). No CD206-positive cells were observed in the HAG treatment group, indicating that HAG alone did not promote the expression of the anti-inflammatory M2 marker CD206 in BV2 cells. In Western blot experiments, we analyzed arginase-1 (Arg-1) and CD206 as typical markers for M2-type BV2 cells (Fig. 2f). The Control and HAG groups did not express Arg-1, though a small amount of CD206 was detected. However, after treatment with IL-4-Biotin HAG, the expression of both Arg-1 and CD206 significantly increased. In conclusion, both immunofluorescence and Western blotting confirmed that IL-4-Biotin HAG can effectively promote BV2 cells polarization towards the M2 phenotype.

### 2.3 DSDH construction and its programmatic modulation for microglia/macrophage polarization

**Fig. 3:**
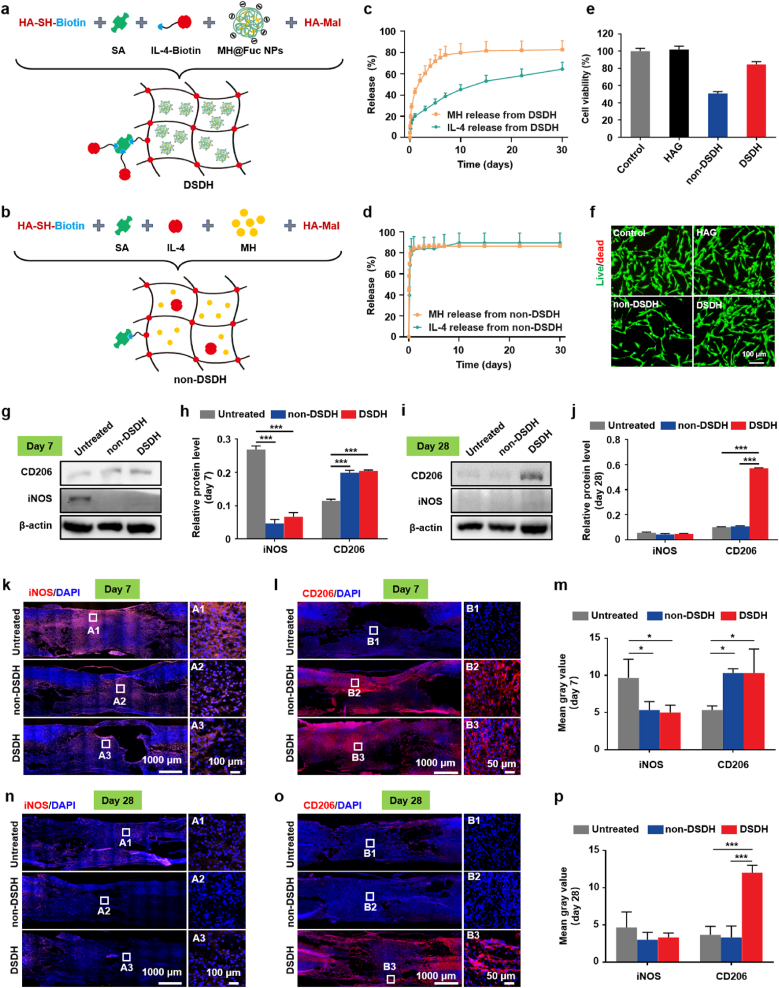
**DSDH construction and its programmatic modulation for microglia/macrophage polarization.** (a–b) Schematic illustration of the DSDH and non-DSDH construction process. (c) Release profiles of MH (n = 3) and IL-4 (n = 4) from DSDH. (d) Release profiles of MH (n = 3) and IL-4 (n = 4) from non-DSDH. (e) Viability and (f) live/dead staining results of PC-12 cells after 72 h of treatment with extracts from DSDH and non-DSDH (n = 3). (g–j) Western blot analysis of iNOS and CD206 expression levels in rats with spinal cord injury (SCI) treated with DSDH and non-DSDH at 7 and 28 days post-treatment (n = 3). (k–l) Immunofluorescence staining of iNOS and CD206 in SCI rats treated with DSDH and non-DSDH at 7 days post-treatment. (m) Quantitative analysis of the average fluorescence intensity for iNOS and CD206 measurements from three animals chosen randomly from each group (n = 3). (n–o) Immunofluorescence staining of iNOS and CD206 in SCI rats at 28 days post-treatment with DSDH and non-DSDH. (p) Quantitative analysis of the mean fluorescence intensity for iNOS and CD206 measurements from three animals chosen randomly from each group (n = 3). Data are presented as mean ± SD and statistical significance was analyzed via one-way ANOVA with Tukey's multiple comparison test; *p < 0.05, ***p < 0.001.

This study developed a DSDH using MH@Fuc NPs, IL-4, a biotin-streptavidin system, HA-SH, and HA-Mal to co-deliver MH and IL-4. As a control, a dual-drug non-sequential delivery hydrogel (non-DSDH) was prepared by directly encapsulating MH and IL-4 into the hydrogel networks (Fig. 3a and b). We have included rheological characterization of both DSDH and non-DSDH formulations to assess the impact of incorporating IL-4 and the biotin-streptavidin system (Fig. S9). The G′ of DSDH is essentially equivalent to that of MH@Fuc HAG (Fig. S6), indicating that the incorporation of IL-4 and the biotin-streptavidin system exerts minimal influence on the rheological properties of the hydrogel. Similarly, the G′ of non-DSDH closely matches that of HAG (Fig. S5c), demonstrating that the introduction of MH, IL-4, and the biotin-streptavidin system likewise has negligible effects on the hydrogel's rheological performance. The results demonstrated that the G′ was not significantly affected by these modifications, indicating that the viscoelastic properties of the hydrogel were well preserved and the mechanical integrity required for spinal cord application remains uncompromised. Both freeze-dried DSDH and non-DSDH exhibited a porous, interconnected microporous structure with pore sizes ranging from 30 to 100 μm (Fig. S10). Notably, MH@Fuc NPs were uniformly distributed on the polymer sheets of the DSDH, while no particles were observed in the non-DSDH. To confirm minimally invasive delivery feasibility, we conducted syringe-injection experiments. HA-Mal solutions containing MH@Fuc NPs and HA-SH solutions incorporating the biotin–streptavidin/IL-4 complex were loaded into a dual-barrel syringe and co-extruded through a static mixer. This process resulted in rapid in situ gelation within seconds to minutes directly at the lesion site, as shown in the Movie S1.

The release profiles of MH and IL-4 from the DSDH were evaluated under simulated physiological conditions using UV–Vis and ELISA, respectively. The results demonstrated that the DSDH exhibited sustained release characteristics for MH: 42.73 % of MH was released within 24 h, and 77.73 % was released by 7 days (Fig. 3c). In contrast, the non-DSDH showed rapid MH release, with 84.06 % released within 24 h, followed by a plateau phase (Fig. 3d). Similarly, the DSDH displayed sustained IL-4 release, with 20.19 % released within 24 h, gradually increasing to 38.76 % by 7 days and 64.41 % by 30 days (Fig. 3c). In contrast, the non-DSDH exhibited rapid IL-4 release, with 83.04 % released within 24 h, followed by a slight increase and eventual plateau (Fig. 3d). These results indicate that the DSDH enables early MH release followed by gradual IL-4 release. The DSDH was constructed by assembling positively charged MH and negatively charged Fuc into MH@Fuc NPs through electrostatic interactions, which were then loaded into the HAG. IL-4 was loaded into the HAG using a stronger biotin-streptavidin affinity system, ensuring the sequential release kinetics of MH and IL-4.

The cytocompatibility of the DSDH was evaluated using PC-12 cells, as biomaterial compatibility is critical for SCI repair. After 72 h of incubation with HAG extracts, PC-12 cell viability was 102.3 %, indicating excellent cytocompatibility of the HAG as a drug delivery carrier (Fig. 3e). The DSDH extract maintained a cell viability of 84.7 %, demonstrating favorable cytocompatibility. In contrast, the non-DSDH group showed a cell survival rate of only 51.0 %, indicating significant cytotoxicity. Live/dead staining further confirmed high cell viability in the control, HAG, and DSDH groups (Fig. 3f). However, the number of viable PC-12 cells was significantly reduced in the non-DSDH group. This difference can be attributed to the slower MH release in the DSDH group, resulting in lower MH concentrations in the extract and reduced cytotoxicity. In contrast, the rapid MH release in the non-DSDH group led to higher MH concentrations and increased cytotoxicity. To verify that the observed cytotoxicity of the non-DSDH extract was indeed attributable to the high initial concentration of MH, we performed an additional control experiment using free MH at an equivalent “burst-release” concentration (12.6 μg/mL) calculated from the non-DSDH release profile (Fig. S11). The results showed that free MH at this concentration induced a comparable reduction in cell viability to that observed with the non-DSDH extract, whereas the other components of the non-DSDH (without MH formulation) extract exhibited no cytotoxic effect. These findings confirm that the cytotoxicity is primarily due to the rapid release of MH rather than other components of the non-DSDH formulation (Fig. S11). These findings suggest that the sustained release mechanism of the DSDH mitigates MH toxicity.

After the programmed release kinetics control of small-molecule drugs and cytokines, and excellent cytocompatibility of the DSDH were confirmed, a suitable disease model was selected to assess the critical in vivo role of dynamic regulation. At SCI site, microglia (resident in the nervous system) and macrophages (infiltrating from the vascular endothelium) regulate the inflammatory environment by secreting various cytokines [17,18]. After SCI, resting microglia/macrophages (M0) quickly polarize into the “classically activated” M1 phenotype, which predominates in the first week after injury [19]. The “alternatively activated” M2 phenotype emerge later, typically after one week, in smaller numbers [19]. MH, a tetracycline antibiotic, effectively inhibits the polarization of microglia/macrophages toward the M1 phenotype, without affecting M2 cells [20]. IL-4 promotes the polarization of microglia/macrophages toward the M2 phenotype by activating signaling pathways such as PI3K/Akt and JAK1/STAT6. When delivered through intramedullary, intraperitoneal, or local administration, IL-4 reduces the accumulation of inflammatory factors at the injury site, enhances tissue regeneration [21]. Because the M1 and M2 polarization responses occur at different times after SCI, the aforementioned programmable delivery system with sequentially releasing of MH and IL-4 capability can be employed to address the dynamics and complexity of microglia/macrophage polarization following SCI. Hence, we further evaluated whether the DSDH could programmatically regulate neuroinflammation using the fully spinal cord crushed model. The SD rats were divided into three groups randomly: (1) the untreated group (SCI without treatment), (2) the non-DSDH group (SCI and treated with non-DSDH), (3) the DSDH group (SCI and treated with DSDH). The phenotypic polarization of microglia/macrophages in different groups were detected on day 7 and 28 by Western blot and Immunofluorescence staining. At 7 days post-treatment, the untreated group showed higher levels of pro-inflammatory iNOS and lower levels of anti-inflammatory CD206 (Fig. 3g and h). In contrast, both the non-DSDH and DSDH groups exhibited reduced iNOS expression and increased CD206 expression. By 28 days, the DSDH group showed significantly higher levels of CD206 compared to the non-DSDH and untreated groups (Fig. 3i and j). Pro-inflammatory iNOS expression was nearly undetectable in all groups at this time point. Immunofluorescence staining of longitudinal spinal cord sections further validated these findings. At 7 days post-SCI, the untreated group displayed numerous iNOS-positive cells and almost no CD206-positive cells. In contrast, the non-DSDH and DSDH groups showed fewer iNOS-positive cells and more CD206-positive cells (Fig. 3k, l, and m). By 28 days, the DSDH group exhibited a higher number of CD206-positive cells compared to the other groups, with minimal iNOS-positive cells observed across all groups (Fig. 3n, o, and p). These results align with the Western blotting data. Therefore, the DSDH's programmatic drug release significantly suppressed pro-inflammatory protein expression at the one-week treatment point, addressing the early dominance of M1-type microglia/macrophages. Additionally, it promoted anti-inflammatory protein expression at the four-week treatment point, compensating for the late-stage scarcity of M2 cells.

To evaluate the released IL-4's bioactivity from DSDH, we assessed the expression of phospho-STAT6 (p-STAT6) in the injury core at 28 days post of injury by immunofluorescence staining. Compared with the untreated and non-DSDH groups, the DSDH group presented a significant increase in phospho-STAT6 fluorescence intensity (Fig. S12). The results clearly demonstrated enhanced p-STAT6 expression in the DSDH treatment group, thereby confirming that the released IL-4 maintained its biological activity and effectively activated canonical signaling pathway in vivo.

### 2.4 DSDH improves hindlimb locomotor and bladder functions in SCI rats

**Fig. 4:**
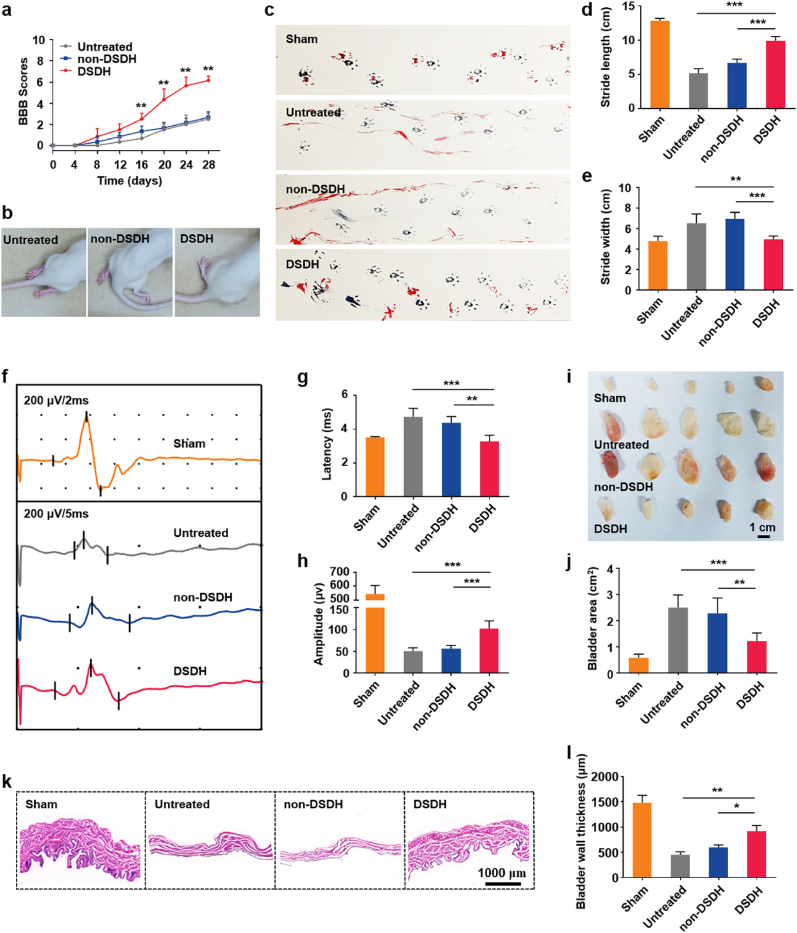
**DSDH improves hindlimb locomotor and bladder functions in SCI rats.** (a) BBB scores. Asterisks represent the comparison of untreated vs DSDH (n = 6). Data are presented as mean ± standard deviation and statistical significance was analyzed via the independent sample *t*-test. **p < 0.01. (b) Recovery of hindlimb motor function at 4 weeks post-surgery. (c) Representative footprint patterns of SCI rats 28 days after treatment with DSDH and non-DSDH. (d) Quantitative analysis of hindlimb stride length (n = 6). (e) Quantitative analysis of hindlimb stride width (n = 6). (f) Motor-evoked potential (MEP) recordings in SCI rats 28 days after treatment with DSDH and non-DSDH. (g) Quantitative analysis of MEP latency (n = 5). (h) Quantitative analysis of MEP amplitude (n = 5). (i) Visual appearance of bladder tissue from SCI rats treated with DSDH and non-DSDH at 28 days post-surgery. (j) Quantitative analysis of bladder tissue area (n = 5). (k) H&E staining of bladder tissue 28 days after treatment with DSDH and non-DSDH. (l) Quantitative analysis of bladder tissue thickness (n = 3). Data are presented as mean ± SD and statistical significance was analyzed via one-way ANOVA with Tukey's multiple comparison test; *p < 0.05, **p < 0.01, ***p < 0.001.

After confirming that the DSDH could programmatically regulate neuroinflammation in SCI area, we investigated whether the DSDH could also improve hindlimb locomotor functions. To assess this, we first evaluated hindlimb performance using the Basso, Beattie, and Bresnahan (BBB) scale (Fig. 4a and b, and Movies S2-S4). Rats in the untreated and non-DSDH groups exhibited only ankle movements, with average BBB scores of approximately 2.5 and 2.7, respectively. In contrast, rats treated with the DSDH showed significant improvement in hindlimb locomotor function, achieving an average BBB score of 6.2 by 28 days, characterized by hindlimb plantar placement. To further evaluate functional recovery, we analyzed hindlimb performance using footprint patterns (Fig. 4c). At 28 days post-SCI, rats in the untreated and non-DSDH groups continued to drag their hindlimbs. In contrast, SCI rats treated with the DSDH demonstrated marked improvement in coordinated locomotor function, displaying regular crawling patterns. Quantitative analysis of footprints revealed that DSDH treatment significantly increased stride length and reduced stride width, indicating improved coordination between the forelimbs and hindlimbs (Fig. 4d and e). We also performed motor-evoked potential (MEP) tests to assess electrophysiological conductivity and neural circuit integrity (Fig. 4f–h). The untreated and non-DSDH groups showed weak electrical conduction recovery, with prolonged latency and low MEP amplitude. In comparison, the DSDH group exhibited significantly enhanced electrophysiological recovery, with reduced latency and increased amplitude.

Bladder dysfunction is a common and severe complication of SCI, often leading to urinary tract infections, bladder stones, urinary incontinence, and, in severe cases, renal failure or bladder cancer, significantly impacting patients' quality of life. At 28 days post-surgery, the bladders of untreated rats exhibited significant stretching, thinning, increased transparency, and congestion compared to the Sham group (Fig. 4i and j). Similar abnormalities were observed in the non-DSDH group. However, DSDH treatment significantly alleviated these bladder abnormalities. Histological analysis using hematoxylin and eosin (H&E) staining further confirmed these findings (Fig. 4k and l). The bladder wall cell density in the DSDH group was relatively high, and the bladder wall thickness was closer to that of normal rats. In contrast, the bladder walls in the untreated and non-DSDH groups were significantly thinner. Quantitative analysis revealed bladder wall thicknesses of 1479 μm (Sham), 453 μm (untreated), 598 μm (non-DSDH), and 918 μm (DSDH). The DSDH group's bladder wall thickness was significantly greater than that of the untreated and non-DSDH groups and closer to the Sham group, indicating that DSDH treatment effectively mitigates pathological damage to bladder muscles.

Histological analysis was conducted to evaluate the SCI lesion area and cavitation status. Longitudinal spinal cord sections, including the lesion area, were stained with H&E (Fig. S13a and S13b). The relative area of the lesion cavity in the DSDH group decreased to 76.62 %, significantly lower than that of the untreated (100 %) and non-DSDH (99.60 %) groups. Pathological examination confirmed that DSDH treatment provided remarkable protection to SCI tissues compared to other treatments, further supporting its efficacy in SCI repair. This protective effect may be attributed to the DSDH's ability to initially release MH and gradually release IL-4, programmatically regulating microglia/macrophage phenotypes at the SCI site. By improving the injury microenvironment and promoting cell growth in the SCI area, the DSDH leads to denser tissue formation and reduces the size of the injury cavity. Additionally, the DSDH may effectively mitigate secondary damage to adjacent spinal tissue, further reducing the cavity ratio in the injury area.

### 2.5 DSDH reduced glial scar deposition and promoted neuron regeneration/survival

**Fig. 5:**
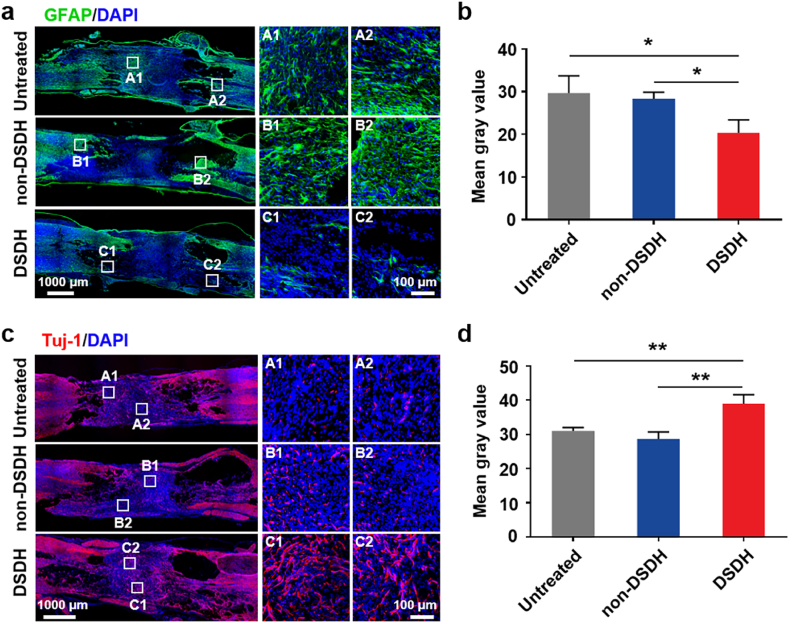
**DSDH reduced glial scar deposition and promotedneuron regeneration/survival.** (a) GFAP expression in the lesion area. (b) Quantification of GFAP-positive astrocytes in the untreated, non-DSDH, and DSDH groups measurements from three animals chosen randomly from each group (n = 3). (c) Tuj-1-positive neurons in the lesion area. (d) Quantification of Tuj-1-positive neurons in the spinal cord from the untreated, non-DSDH, and DSDH groups measurements from three animals chosen randomly from each group (n = 3). Data are presented as mean ± SD and statistical significance was analyzed via one-way ANOVA with Tukey's multiple comparison test; *p < 0.05, **p < 0.01.

Following SCI, an explosive increase in activated glial cells accumulates at the border between injured and normal tissue, leading to glial scar formation. This scar tissue disrupts the self-repair ability of spinal cord tissues due to persistent inflammation. After confirming that the DSDH could programmatically regulate microglia/macrophage polarization in vivo and improve hindlimb locomotor recovery post-SCI, we investigated whether DSDH treatment could mitigate neuroinflammation-induced glial scar formation. To this end, we evaluated the expression of glial fibrillary acidic protein (GFAP), a biomarker for astrocytes, in the lesion area of SCI rats after different treatments (Fig. 5a and b). In the untreated and non-DSDH groups, abundant GFAP expression was observed, indicating a significant increase in GFAP-positive cells. However, this abnormality was markedly reduced in the DSDH-treated group. These findings suggest that the DSDH may inhibit glial scar formation.

Improving the inflammatory microenvironment is critical for protecting neurons and promoting the generation of new neurons. The results above demonstrate that the DSDH can programmatically regulate SCI-induced neuroinflammation. Therefore, we further investigated the impact of DSDH on neurons at the SCI site. Tuj-1 (β-III-tubulin), a protein expressed in neurons—primarily in axons and dendrites—is commonly used as an early neuronal marker. It plays a significant role in neuronal formation, migration, and differentiation. Weaker Tuj-1-positive neurons were observed in the lesion areas of the untreated and non-DSDH groups (Fig. 5c). In contrast, stronger Tuj-1-positive neurons were detected in the DSDH-treated group. Quantitative analysis of the percentage area occupied by Tuj-1-positive sites revealed a significantly higher percentage in the DSDH group compared to the untreated and non-DSDH groups (Fig. 5d).

Neurofilaments regeneration and synapse formation are crucial for functional recovery. To evaluate the extent of neurofilaments regeneration and synapse formation, we performed immunostaining for neurofilaments (NFs) and the presynaptic marker synapsin I at 4 weeks post injury. Compared with the untreated and non-DSDH groups, DSDH group presented a significant increase in NFs (Fig. S14) and synapsin I (Fig. S15) fluorescence intensity. The results revealed that the DSDH-treated group exhibited extensive neurofilaments regeneration and synaptic reconstruction, highlighting its ability to promote neuronal connectivity and axonal integrity.

Biocompatibility is a critical factor for therapeutic biomaterial systems. To evaluate this, we conducted histological analysis of key organs—including the heart, liver, spleen, lung, and kidney—using H&E staining after 28 days of treatment (Fig. S16). No obvious pathological abnormalities or lesions were observed in the tissues of any group. These results indicate that the DSDH did not significantly affect the key organs or cause systemic toxicity after 28 days of treatment, demonstrating its favorable biocompatibility.

## 3 Discussions and conclusion

SCI is a devastating condition that leads to severe sensory and motor dysfunction, often resulting in permanent disability [13,14]. The complex inflammatory response following SCI, characterized by the dynamic polarization of microglia/macrophages into pro-inflammatory M1 and anti-inflammatory M2 phenotypes, plays a critical role in the secondary injury process [15]. Several biomaterial-based strategies have been explored for SCI treatment by targeting microglia/macrophage polarization, either by inhibiting M1 polarization or promoting M2 polarization [24,34,35]. In a previous study, we used a metal coordination method combined with polymeric networks to locally deliver minocycline to SCI lesions. The slight acidity in the injured area triggered minocycline release, inhibiting microglia/macrophage polarization toward the M1 phenotype [24]. Xi et al. developed electrospun fiber scaffolds grafted with liposomes containing a plasmid encoding IL-4, linked through Schiff base bonds. These scaffolds released the plasmid-loaded liposome in SCI area, inducing M2 polarization and the secretion of anti-inflammatory factors from microglia/macrophages [34]. Another study showed that the anti-inflammatory cytokine IL-10, immobilized in a visible light-crosslinked gelatin hydrogel, promoted the M2 phenotype and supported nerve regeneration in a SCI mouse model [35]. While M1 polarization dominates the initial acute stage of injury, exacerbating inflammation and tissue damage, M2 polarization emerges in a sequential manner during the non-acute stage, promoting tissue repair and functional recovery [19]. However, previous reports have not resolved the challenge of managing the temporal dynamics between these polarization states.

In this study, we developed a dual-drug sequential delivery hydrogel (DSDH) system designed to address these dynamic inflammatory processes following SCI. Using MH to inhibit M1 polarization during the acute inflammatory stage and IL-4 to promote M2 polarization during sequential non-acute stage, our DSDH system effectively modulated the inflammatory microenvironment. This temporal regulation of microglia/macrophage polarization provided significant therapeutic benefits, as evidenced by improvements in functional recovery, reduced secondary damage, and enhanced tissue repair in SCI rats, compared to non-programmed delivery systems. A key advantage of the DSDH is its ability to release MH and IL-4 in a controlled, sequential manner.

The release profiles of MH and IL-4 confirmed this sequential drug delivery, with MH released during the first 7 days, and IL-4 gradually released over 30 days (Fig. 3). In vivo studies demonstrated that the DSDH effectively suppressed M1 polarization in the early stages of SCI while promoting M2 polarization in the later recovery phase (Fig. 3). These findings emphasize the importance of temporal drug release in addressing the dynamic nature of SCI pathology. In terms of functional outcomes, rats treated with DSDH showed significant improvements in hindlimb locomotion, bladder function, and tissue repair. Behavioral assessments revealed marked recovery, as evidenced by improved BBB scores, footprint analysis, and MEP tests (Fig. 4). Histological analyses also showed reduced lesion cavity size, better tissue integrity, and enhanced Tuj-1^+^ neuron regeneration and survival (Fig. 5, Fig. S13). These results suggest that DSDH not only modulated the inflammatory response but also promoted tissue remodeling and neuronal survival, both essential for SCI recovery. Importantly, the DSDH system was biocompatible, showing no significant toxicity in key organs like the heart, liver, spleen, lungs, and kidneys, which is crucial for its potential clinical application (Fig. S16).

Based on the drug release profiles (Fig. 3c and d), the release model indicates that the two drugs reach their plateau at different times. Once MH reaches its peak, its release rate decreases to a relatively low level, while IL-4 continues to be released. This pattern allows the drugs to exert their effects in succession, making this a form of sequential release. In this model, the cumulative release of IL-4 is generally lower than that of MH. The efficacy of the dual-drug treatment is higher than that of monotherapy, due to the synergistic effects of both drugs. However, one limitation of this release model is that the simultaneous release of both drugs, as often seen in early release stage due to difficulties in ensuring absolute binding or preventing interference with the drugs’ interaction with the carrier [25]. As shown in the release profiles (Fig. 3c and d), a small fraction of IL-4 is indeed released during the first week, overlapping with MH release. However, our in vivo data at 7 days post-injury (Fig. 3g, h, 3k-3m) demonstrated that DSDH treatment effectively suppressed M1 microglia/macrophage polarization during the acute phase, indicating that this early IL-4 release did not interfere with the anti-inflammatory activity of MH. Instead, the sustained presence of IL-4 subsequently promoted M2 polarization at later stages (Fig. 3i, j, 3n-3p), which aligns with the therapeutic goal of spatiotemporal immune modulation. Furthermore, as demonstrated by our cytotoxicity assays, uncontrolled and rapid MH release from non-DSDH led to measurable toxicity, whereas DSDH exhibited no significant cytotoxic effects despite releasing ∼40 % MH within the first 24 h (Fig. 3c). This suggests that the release profile from DSDH, though relatively fast initially, remains within a tolerable range for local cells. The spatiotemporally controlled release design of DSDH likely mitigates potential toxicity by distributing MH release more gradually compared with non-DSDH. Nevertheless, we will further fine-tune of the release profile could be beneficial in the future. Strategies such as modifying hydrogel crosslinking density or tailoring nanoparticle-matrix interactions will further slow MH release and optimize the therapeutic window.

In our previous work, we systematically investigated MH-loaded hydrogels for SCI treatment and demonstrated that MH alone effectively suppressed M1 polarization and partially improved functional recovery [24]. Likewise, other studies have reported that IL-4 delivery alone promotes M2 polarization and enhances motor recovery after SCI [36]. Building on these established findings, our current study was designed to focus specifically on the sequential and programmable release strategy enabled by DSDH, rather than to re-evaluate single-agent effects. Importantly, our data show that although the non-DSDH formulation transiently inhibited M1 polarization, only DSDH achieved spatiotemporal immunomodulation—suppressing M1 polarization during the acute phase and subsequently promoting M2 polarization during the chronic phase—ultimately leading to superior functional recovery. These results support the necessity of dual-drug sequential delivery. We acknowledge that including additional control groups-MH-only hydrogel, IL-4-only hydrogel, and an empty HA hydrogel-would further clarify the individual and synergistic contributions of each component. We will extend the above control groups in future work to further strengthen the mechanistic conclusions.

The selected dose of 300 μg MH was based on our prior study [24], where this dosage significantly suppressed M1 polarization, attenuated inflammatory responses, and promoted functional recovery without signs of toxicity. Using the same dose here ensured consistency and comparability with these established results. For IL-4, we selected 500 ng per rat based on prior reports in SCI models [36], which demonstrated that nanogram-level dosing was effective. Importantly, IL-4 was delivered through our DSDH system to achieve sustained release within the lesion core, maintaining a therapeutic local concentration while minimizing systemic exposure. We plan to extend the formal dose-response study in future studies to address the dosing concerns.

In the present study, our primary focus was on demonstrating the programmed regulation of microglia/macrophage phenotypes via DSDH and its therapeutic relevance in SCI. Previous studies have already established that M2-polarized microglia and macrophages secrete elevated levels of BDNF and GDNF, which contribute to neuronal survival and axonal regeneration [37,38]. On this basis, we did not further investigate neurotrophic factor secretion in the current work. We will focus on quantifying neurotrophic factor release and dissecting the downstream signaling pathways by which M2 cells support neuroregeneration in the future work.

Regarding the issues of DSDH degradation kinetics and potential long-term inflammatory responses, as shown in Fig. S16, histological examination of major organs revealed no acute toxicity, supporting the short-term biosafety of DSDH. Preliminary in vivo observations up to 28 days also showed no signs of persistent inflammation at the lesion site (Fig. S13), suggesting a favorable local response. Nevertheless, we acknowledge that systematic investigation of hydrogel degradation kinetics and potential long-term inflammatory effects was not performed in the present study. We plan to extend our investigations in future studies by incorporating long-term degradation analyses, immunohistochemical assessments, and inflammatory cytokine profiling to comprehensively assess chronic biocompatibility and safety.

In conclusion, the DSDH system represents a promising therapeutic approach for SCI by providing a temporal control mechanism for microglia/macrophage polarization. This dual-drug, sequential release strategy not only regulates neuroinflammation but also enhances functional recovery, reduces secondary damage, and promotes tissue repair. Future studies will aim to refine the system's delivery kinetics, explore alternative drug combinations, and investigate the long-term effects of DSDH treatment on SCI recovery. Additionally, the system's potential for application in other neuroinflammatory conditions, such as Alzheimer's disease and stroke, warrants further exploration, as it may offer broader therapeutic benefits for treating neurodegenerative diseases.

## 4 Materials and methods

### 4.1 Materials

Hyaluronic acid (HA, ∼200 kDa) was purchased from Bloomage Biotechnology Co., Ltd. (Jinan, China). Fluorescein isothiocyanate (FITC), Fuc, BSA, Lys, SA, and MH were obtained from Sigma-Aldrich Co., Ltd. (USA), Macklin Reagent Co., Ltd. (Shanghai, China), Beijing Solarbio Science & Technology Co., Ltd. (Beijing, China), and Meilun Biotech. (Dalian, China), respectively. Biotin-PEG-Mal and biotin-NHS were purchased from Ponsure Biotechnology (Shanghai, China) and MedChemExpress (USA), respectively. IL-4 was acquired from PeproTech (USA), and the IL-4 ELISA Kit was obtained from Multisciences (Lianke) Biotech Co., Ltd. (Hangzhou, China). Cell Counting Kit-8 (CCK-8) and the Calcein/PI Cell Viability/Cytotoxicity Assay Kit were purchased from Bestbio (Shanghai, China) and Beyotime (Shanghai, China), respectively. Phosphate-buffered saline (PBS) and 4 % paraformaldehyde fixative were procured from Servicebio Biotechnology Co., Ltd. (Wuhan, China). Optimum Cutting Temperature (OCT) compound was obtained from Sakura Finetek, Inc. (USA). The primary antibodies used in the research including anti-iNOS antibody (Abcam, ab178945, 1:1000), anti-CD206 antibody (Abcam, ab64693, 1:1000) anti-GFAP antibody (Abcam, ab7260, 1:1000), anti-Tuj-1 antibody (Abcam, ab18207, 1:1000), anti-Neurofilament heavy polypeptide antibody (Abcam, ab207176, 1:1000), anti-Arg-1 antibody (Proteintech, 16001-1-AP, 1:1000), anti-β-actin antibody (Proteintech, 20536-1-AP, 1:1000), Anti-Synapsin I antibody (Selleck, G18B13, 1:500) and Anti-Phospho-STAT6 antibody (Selleck, A16N6, 1:500) were purchased from Abcam (USA), Proteintech (Wuhan, China) and Selleck (USA), respectively. Secondary antibodies including Alexa Fluor 546-conjugated Goat anti-Rabbit Secondary Antibody (Invitrogen, A-11035, 1:1000) and Alexa Fluor 488-conjugated Goat anti-Rabbit Secondary Antibody (Invitrogen, A-11008, 1:1000) were purchased from Invitrogen (USA). HA-SH and HA-Mal were synthesized according to previously reported methods [39,40].

### 4.2 Preparation and characterization of MH@Fuc NPs

MH@Fuc NPs were prepared by mixing Fuc and MH. Briefly, Fuc (60 mg/mL) and MH (5 mg/mL) solutions were prepared in double-distilled water (ddH2O). To form MH@Fuc NPs, 1 mL of Fuc solution was mixed with 800 μL of MH solution and sonicated for 5 min. The mixture was then centrifuged twice at 13,000 rpm for 10 min to isolate the MH@Fuc NPs. The supernatant was collected, and the MH concentration was determined using a UV–Vis spectrometer. Standard curves were used to calculate the MH concentration and mass. The EE and LC of MH@Fuc NPs were calculated using the following formulas:

$$\text{EE}\%=\left({\text{W}}_{\text{m}}-{\text{W}}_{\text{f}}\right)/{\text{W}}_{\text{m}}\times 100\%$$

$$\text{LC}\%=\left({\text{W}}_{\text{m}}-{\text{W}}_{\text{f}}\right)/{\text{W}}_{\text{a}}\times 100\%$$

Where W_m_ is the total mass of MH added, W_f_ is the mass of free MH in the supernatant, and W_a_ is the mass of MH@Fuc NPs.

The hydrodynamic size and zeta potential of MH@Fuc NPs were measured using DLS. FT-IR spectra of MH@Fuc NPs were recorded using an FT-IR spectrometer with the KBr pellet technique.

### 4.3 Preparation and characterization of MH@Fuc HAG

20 mg/mL of MH@Fuc NPs were dispersed in the 2 % (w/v) of HA-Mal solution prior to mixing, thereafter the mixture was mixed with the equal volumes of 2 % (w/v) of HA-SH to obtain MH@Fuc HAG. For, MH HAG, same drug concentration MH was directly loaded into HAG without using MH@Fuc NPs. The microstructures MH@Fuc HAG were examined using SEM (Tescan Mira microscope, Czech Republic). To evaluate the release profile of MH, 100 μL of MH HAG or MH@Fuc HAG was incubated in 1 mL of PBS at 37 °C with gentle shaking at 50 rpm. At predetermined time points (1, 2, 4, 8, 24, 48, 72, 96, 120, 144, and 168 h), the supernatant was replaced with fresh PBS, and the absorbance of the collected supernatant was measured at 244 nm using a UV–Vis spectrometer. The MH concentration was calculated using standard curves.

### 4.4 Preparation and characterizations of protein drug delivery platform

Protein-Biotin conjugates (including IL-4, BSA, and Lys) were prepared by grafting Biotin-NHS onto the amino groups of the proteins. Briefly, protein solutions were prepared by dissolving the proteins in ddH_2_O at 4 °C, while Biotin-NHS was dissolved in dimethyl sulfoxide (DMSO) at room temperature. The protein and Biotin-NHS solutions were mixed at a molar ratio of 1:5 and allowed to react for 0.5 h at 4 °C to form Protein-Biotin conjugates.

For fabricate IL-4-Biotin HAG, HA-SH and Biotin-PEG-Mal solutions were first mixed and reacted for 0.5 h at room temperature to produce Biotin-PEG-Mal-labeled HA-SH (HA-SH-Biotin). Then HA-SH-Biotin was then incubated with streptavidin for 0.5 h at room temperature, followed by incubation with IL-4-Biotin to form HA-SH-Biotin/IL-4 Biotin complexes. IL-4-Biotin HAG was formed by adding HA-Mal to the HA-SH-Biotin/IL-4-Biotin mixture. As a control, IL-4 HAG was prepared using non-biotinylated IL-4 following the same procedure. To evaluate the release profile of IL-4, 100 μL of IL-4 HAG or IL-4-Biotin HAG was incubated in 1 mL of PBS at 37 °C with gentle shaking at 50 rpm. The supernatant was replaced with fresh PBS at predetermined time points (3 h, 6 h, 12 h, 1 d, 3 d, 5 d, 7 d, 10 d, 15 d, 22 d, and 30 d), and the amount of IL-4 released was quantified using an ELISA kit.

### 4.5 Preparation and characterizations of DSDH

MH@Fuc NPs were resuspended in HA-Mal solution to form HA-Mal/MH@Fuc NPs. Based on the preparation method of IL-4-Biotin HAG, DSDH was formed by adding HA-Mal/MH@Fuc NPs dispersion to HA-SH-Biotin/IL-4-Biotin complexes. A non-programmed control system (non-DSDH) was prepared using MH and non-biotinylated IL-4 following the same procedure. Dual-drug release profiles from DSDH and non-DSDH were monitored for 30 days.

To evaluate the biocompatibility of DSDH and non-DSDH, PC-12 cells were cultured in Roswell Park Memorial Institute 1640 (RPMI-1640) medium supplemented with 10 % fetal bovine serum (FBS) and 1 % penicillin-streptomycin at 37 °C in a 5 % CO_2_ incubator. Cell viability was assessed using the CCK-8 assay. Briefly, PC-12 cells (3 × 10^3^ cells/well) were cultured with extracts from DSDH and non-DSDH for 72 h. After removing the culture medium, RPMI-1640 containing 10 % CCK-8 solution was added to each well and incubated for 2 h. Absorbance was measured at 450 nm using a microplate reader. Cell viability was further confirmed using the LIVE/DEAD Viability/Cytotoxicity Kit. PC-12 cells treated with DSDH and non-DSDH extracts for 72 h were stained with Calcein/PI for 30 min at 37 °C and imaged using an inverted fluorescence microscope (Nikon, Japan).

### 4.6 In vitro cellular polarization experiments

To evaluate the effects of MH@Fuc HAG on microglial polarization, BV2 cells were cultured and treated with drug-releasing solutions of HAG, MH HAG, and MH@Fuc HAG for 24 h in the presence of LPS. Microglial phenotype transition was assessed using immunofluorescence staining and Western blotting for iNOS. To evaluate the effects of IL-4-Biotin HAG on microglial polarization, BV2 cells were treated with IL-4-Biotin HAG for 24 h. Microglial phenotype transition was analyzed using immunofluorescence staining and Western blotting for CD206 and Arg-1.

For immunofluorescence staining, cells were fixed with 4 % paraformaldehyde for 15 min, permeabilized with 0.1 % Triton X-100 in PBS for 15 min, and blocked with 5 % FBS for 1 h. Cells were then incubated with primary antibodies (e.g., iNOS) overnight at 4 °C, washed with PBS, and stained with secondary antibodies. Images were acquired using a confocal laser scanning microscope (CLSM, TI-E + A1RMP + N-STORM, Nikon, Japan).

For Western blotting, cells were lysed in passive lysis buffer containing phenylmethylsulfonyl fluoride (PMSF) and protease inhibitor cocktail. Protein concentrations were quantified using a protein assay kit (Applygen Technologies Inc.). Proteins were separated by 10 % SDS-PAGE, transferred to polyvinylidene fluoride (PVDF) membranes, and probed with primary antibodies (e.g., iNOS, CD206, β-actin) overnight at 4 °C. Membranes were incubated with HRP-conjugated secondary antibodies for 1 h at room temperature, and protein expression was visualized using an automatic gel imaging system. Quantification was performed using ImageJ software. The gray value of β-actin was used to normalize the gray value of iNOS and CD206.

### 4.7 SCI rat model and DSDH transplantation

Animal experiments were approved by the Institutional Animal Care and Use Committee of Hunan University (approval number HNUBIO202201002). A SCI model was established using female Sprague-Dawley (SD) rats (190–210 g). The SD Rats were randomly divided into four groups (12 rats per group) including sham, untreated, non-DSDH, and DSDH group, and all rats were acclimatized for 1 week before surgery. For the untreated groups, the spinal cord was fully crushed for 10 s at the T9 level using forceps (3 mm width) under sodium pentobarbital anesthesia (50 mg/kg, intraperitoneal injection). The DSDH and non-DSDH groups underwent the same surgical procedure and received in situ injections of hydrogels (50 μL) containing 300 μg MH and 500 ng IL-4. The sham group underwent the same surgical procedure without SCI and drug administration. Post-surgery, rats were sutured, labeled, and monitored for recovery. Artificial micturition was performed to ensure their well-being twice daily until automatic bladder voiding was restored.

### 4.8 Locomotion behavioral assessment

Motor function recovery was evaluated using the BBB locomotor rating scale and footprint analysis. Two skilled examiners blinded to examine BBB assay. For footprint analysis, forelimbs and hindlimbs were marked with blue and red dyes, respectively. Rats were allowed to walk through a tunnel lined with white paper, and stride length and width were measured from three consecutive gait cycles according to our previous report [41]. MEPs were recorded in the fourth week post-surgery. Rats were anesthetized, and stimulating electrodes were placed in the upper jaw and brain region. A concentric needle electrode was inserted into the gastrocnemius muscle to record MEP signals following electrical stimulation (20 mA).

### 4.9 Tissue histology assessments

At 7 and 28 days post-injury, rats were perfused with PBS and 4 % paraformaldehyde. Spinal cord and organ tissues were fixed, dehydrated, embedded in OCT compound, and sectioned (12 μm thickness). Sections were stained with H&E or processed for immunofluorescence using primary and secondary antibodies. Images were acquired using a confocal laser scanning microscope (Nikon, TI-E + A1RMP + N-STORM, Japan) or a digital slide scanner (Pannoramic MIDI, 3DHISTECH, Hungary). Mean gray value, representing the average fluorescence intensity of the image, was quantified using ImageJ software.

### 4.10 Statistical analysis

Data were analyzed using GraphPad Prism 6.0. Comparisons between two groups were performed using the independent sample *t*-test, while multiple groups were compared using one-way ANOVA followed by Tukey's post-hoc test. Data are presented as mean ± standard deviation. Significance levels were set at *p < 0.05, **p < 0.01, and ***p < 0.001.

## CRediT authorship contribution statement

**Ya Li:** Writing – original draft, Visualization, Validation, Supervision, Methodology, Investigation, Formal analysis, Data curation, Conceptualization. **Yuyun Liang:** Investigation, Formal analysis. **Chaoyong He:** Data curation. **Runxiang Yao:** Investigation. **Ke Jian:** Investigation. **Liyang Shi:** Writing – review & editing, Supervision, Project administration, Investigation, Funding acquisition, Conceptualization.

## Declaration of competing interest

The authors declare that they have no known competing financial interests or personal relationships that could have appeared to influence the work reported in this paper.

## Data Availability

Data will be made available on request.
