# Supplementary material for: A dual-drug sequential delivery hydrogel for programmatic microglia/macrophage polarization and function recovery in spinal cord injury
Source: Mater Today Bio. 2025 Sep 29;35:102365. doi: 10.1016/j.mtbio.2025.102365 (PMC12513293; doi:10.1016/j.mtbio.2025.102365)
Supplement: Multimedia component 1 [file mmc1.docx]

**Supplementary Material:**

**A Dual-drug Sequential Delivery Hydrogel for Programmatic Microglia/Macrophage Polarization and Function Recovery in Spinal Cord Injury**

Ya Li ^1,2#^, Yuyun Liang^1#^, Chaoyong He^1^, Runxiang Yao^1^, Ke Jian^1^, Liyang Shi^1^*

^1^College of Biology, Hunan University, Changsha, 410082, China

^2^Institutes of Health Central Plain, Clinical Medical Center of Tissue Engineering and Regeneration, Xinxiang Medical University, Xinxiang, 453003, China

^*^Corresponding author Liyang Shi, email: liysh777@hnu.edu.cn

# These authors contributed equally to this work.


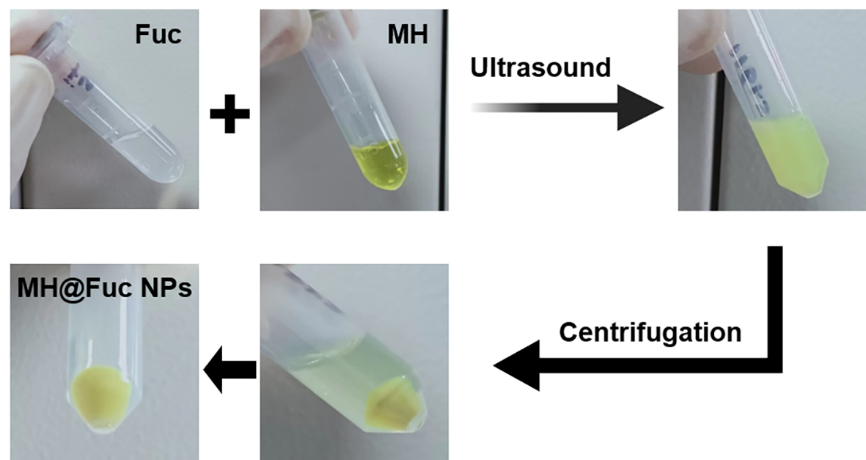


**Figure S1.** Preparation MH@Fuc NPs by mixing Fuc and MH.


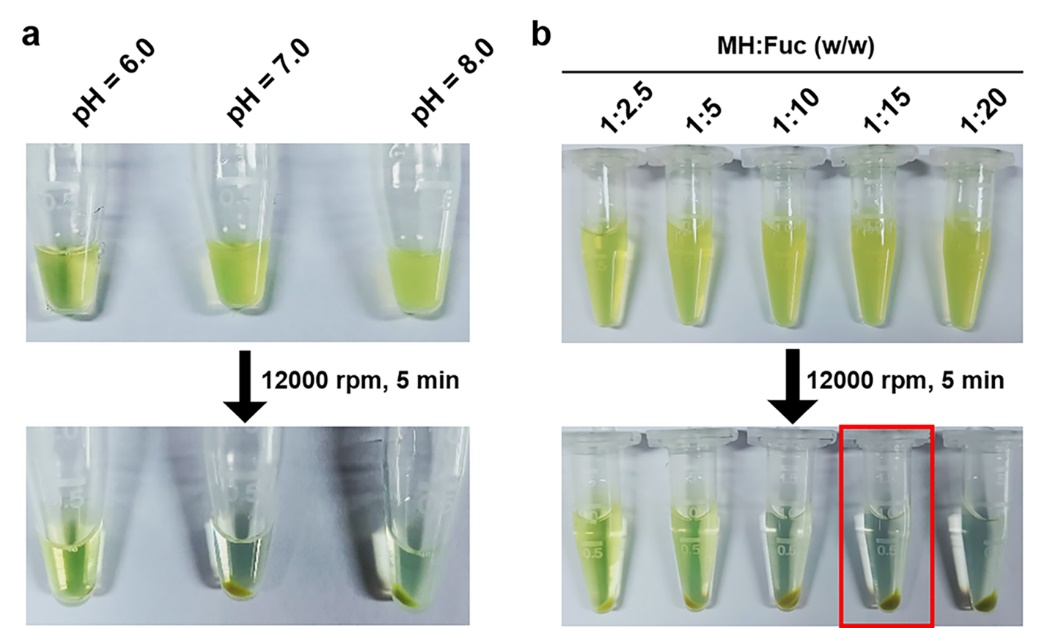


**Figure S2.** (**a**) Formation of MH@Fuc NPs under various pH conditions. (**b**) MH@Fuc NPs were prepared by varying the mass ratio of MH and Fuc.


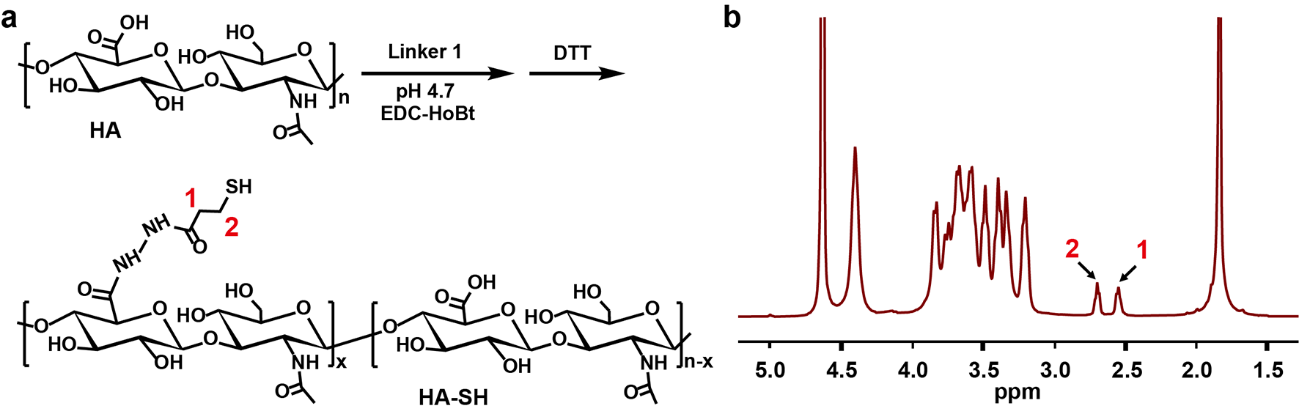


**Figure S3.** (**a**) Synthetic scheme of HA-SH. (**b**) ^1^H-NMR spectrum curve of HA-SH.


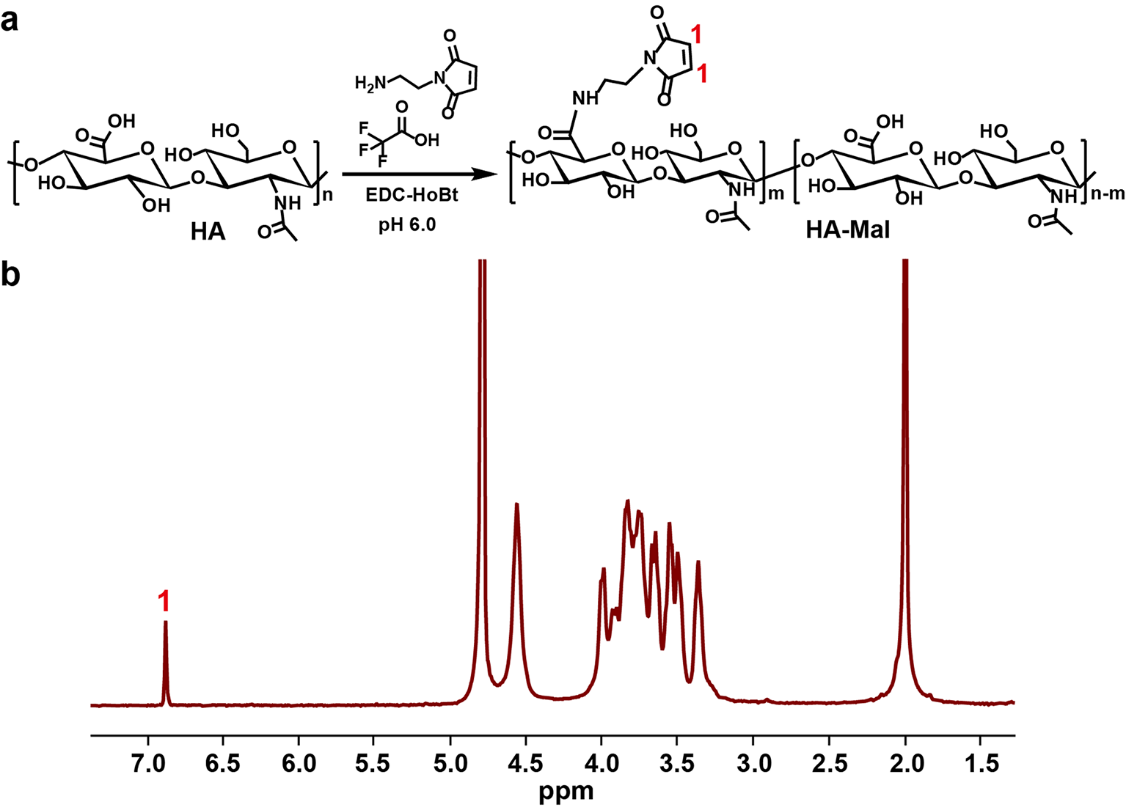


**Figure S4.** (**a**) Synthetic scheme of HA-Mal. (**b**) ^1^H-NMR spectrum curve of HA-Mal.


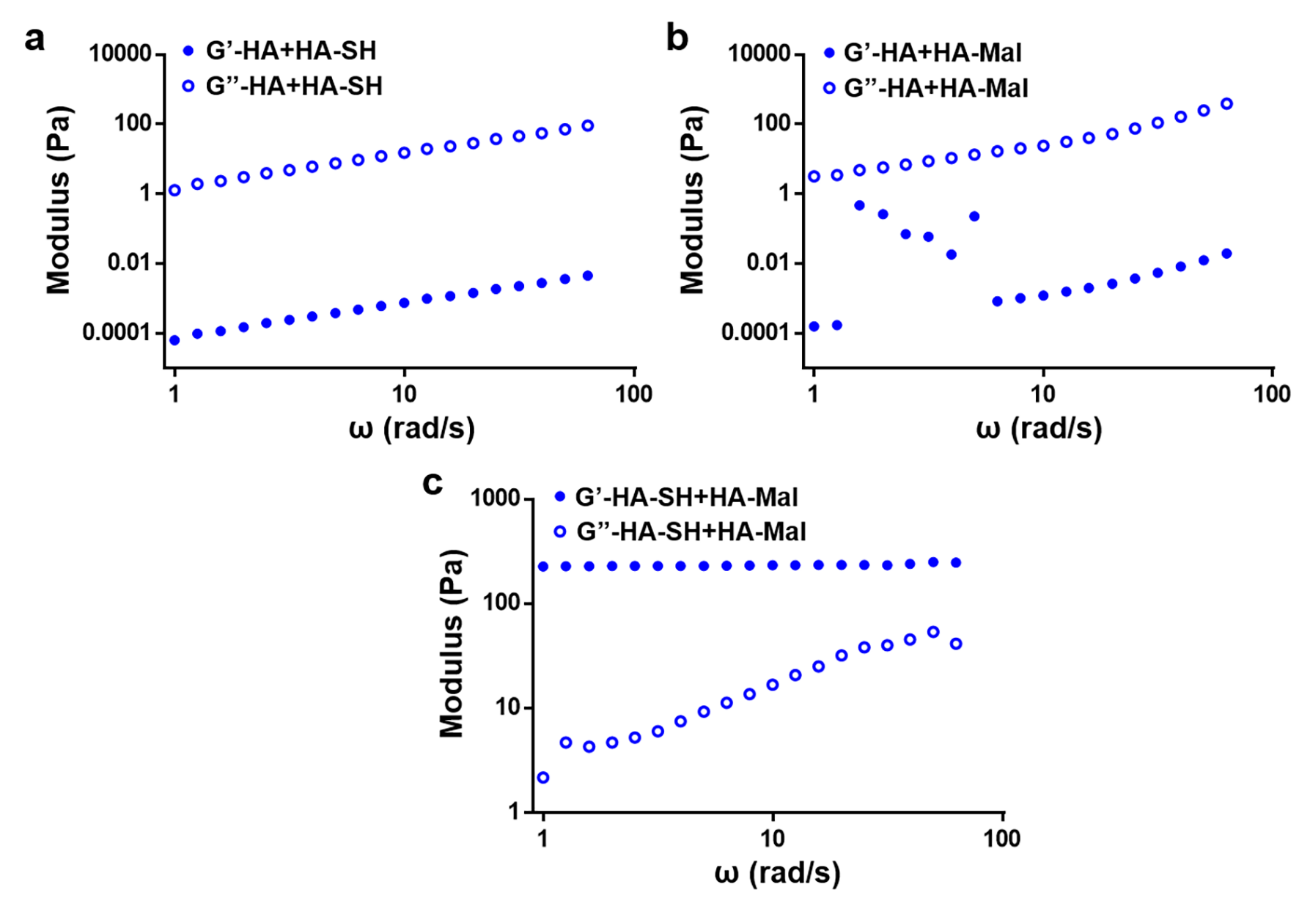


**Figure S5.** G’ and G” of (**a**) the mixture of HA and HA-SH, (**b**) the mixture of HA and HA-Mal, and (**c**) the mixture of HA-SH and HA-Mal as a function of angular velocity.


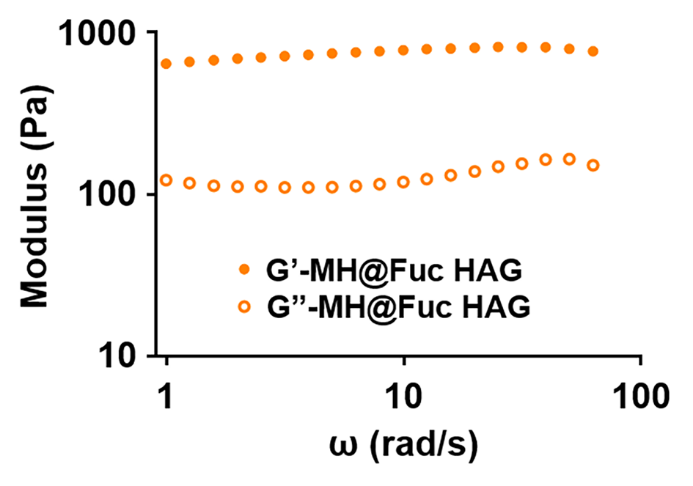


**Figure S6.** G’ and G” of MH@Fuc HAG as a function of angular velocity.


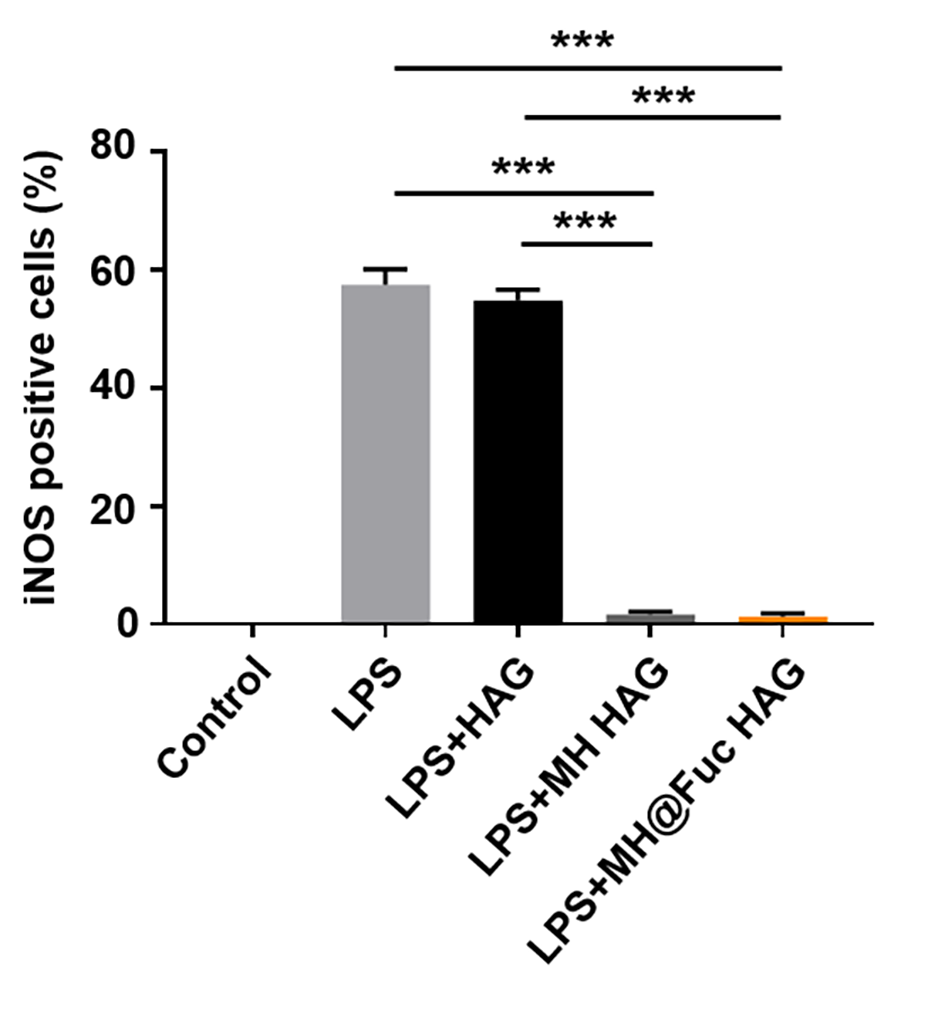


**Figure S7.** iNOS-positive cell percentages after BV2 cells treated by the drug-releasing solution of HAG, MH HAG and MH@Fuc HAG for 24 hours in the presence of LPS (n = 3). Data are presented as mean ± SD and statistical significance was analyzed via one-way ANOVA with Tukey’s multiple comparison test; ***p < 0.001.


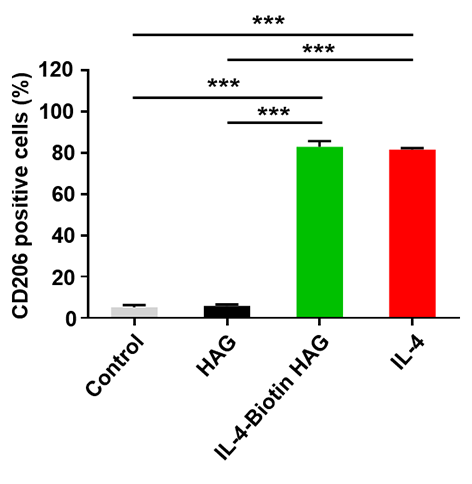


**Figure S8.** CD206-positive cell percentages after BV2 cells treated by the HAG, IL-4-Biotin HAG and IL-4 for 24 hours (n = 3). Data are presented as mean ± SD and statistical significance was analyzed via one-way ANOVA with Tukey’s multiple comparison test; ***p < 0.001.


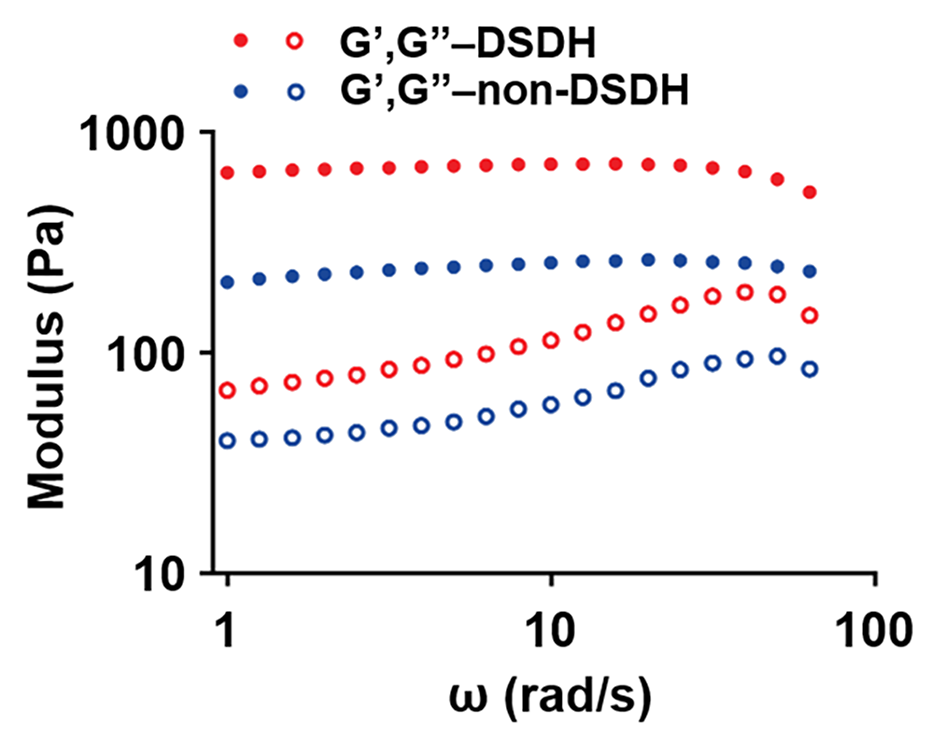


**Figure S9.** G’ and G” of DSDH and non-DSDH as a function of angular velocity.


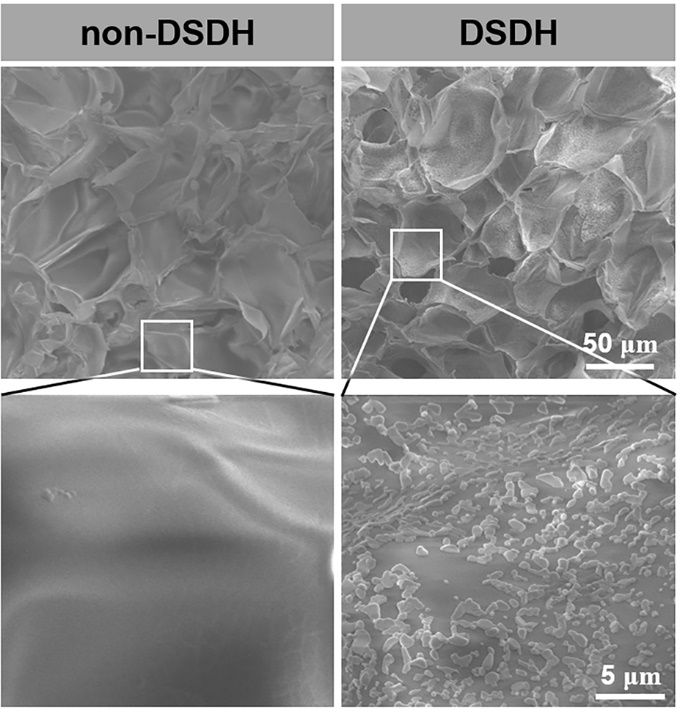


**Figure S10.** Microscopic morphology of freezing-dried non-DSDH and DSDH.


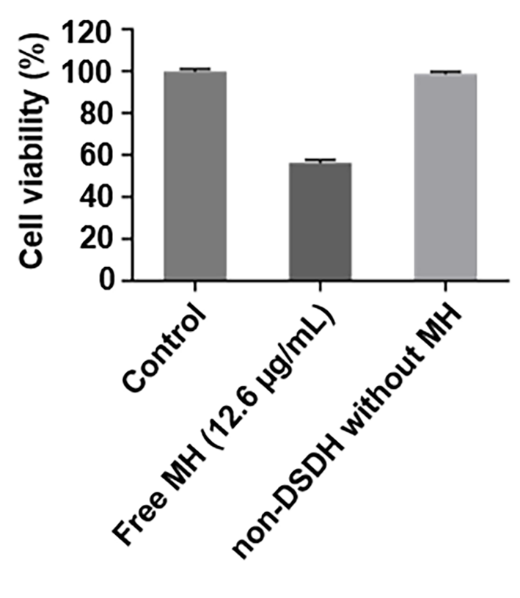


**Figure S11.** Viability of PC-12 cells after 72 hours of treatment with free MH or extracts from non-DSDH without MH (n=4).


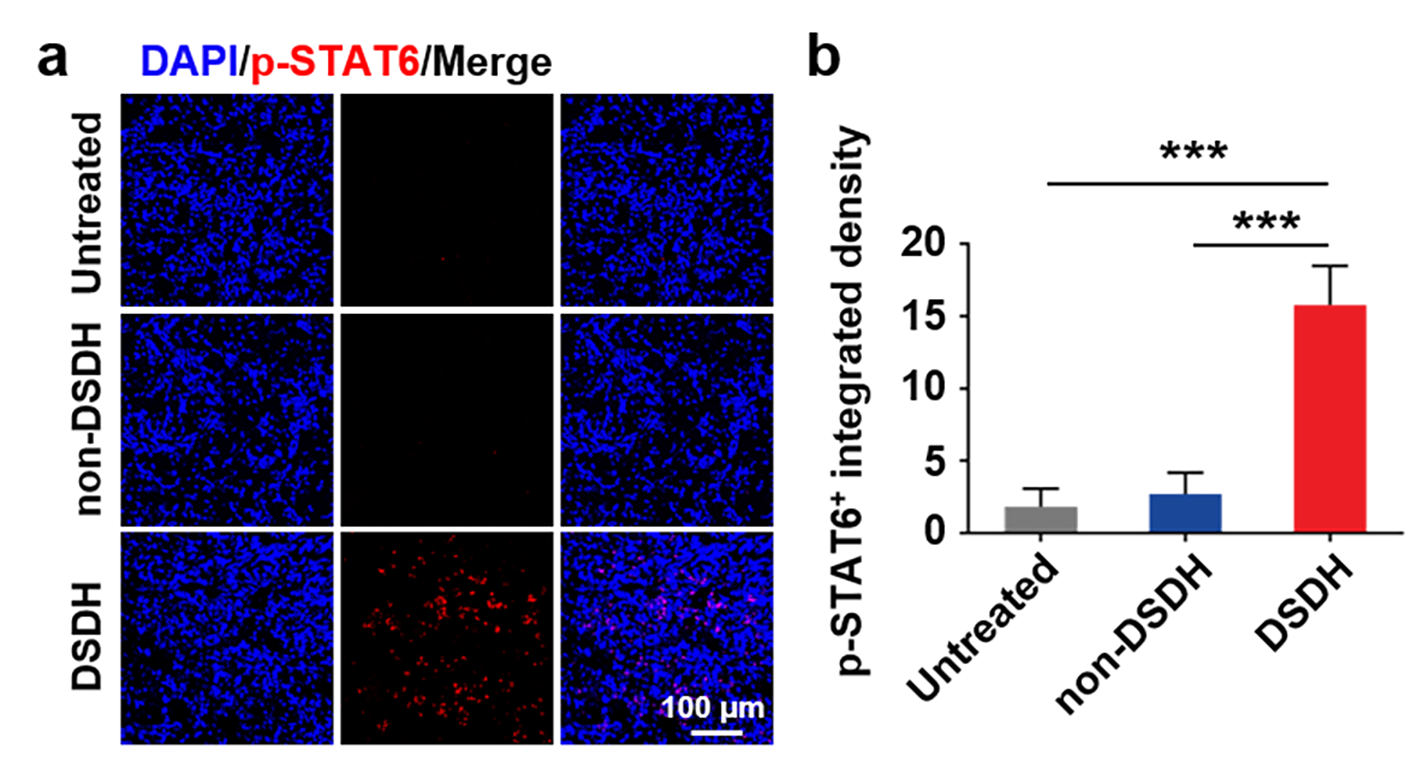


**Figure S12.** Immunofluorescence staining confirms that DSDH promotes the expression of phospho-STAT6 (p-STAT6). (a) p-STAT6 expression in the lesion area. (b) p-STAT6 integrated density in the injury site of the untreated, non-DSDH, and DSDH groups (n = 3). Data are presented as mean ± SD and statistical significance was analyzed via one-way ANOVA with Tukey’s multiple comparison test; ***p < 0.001.


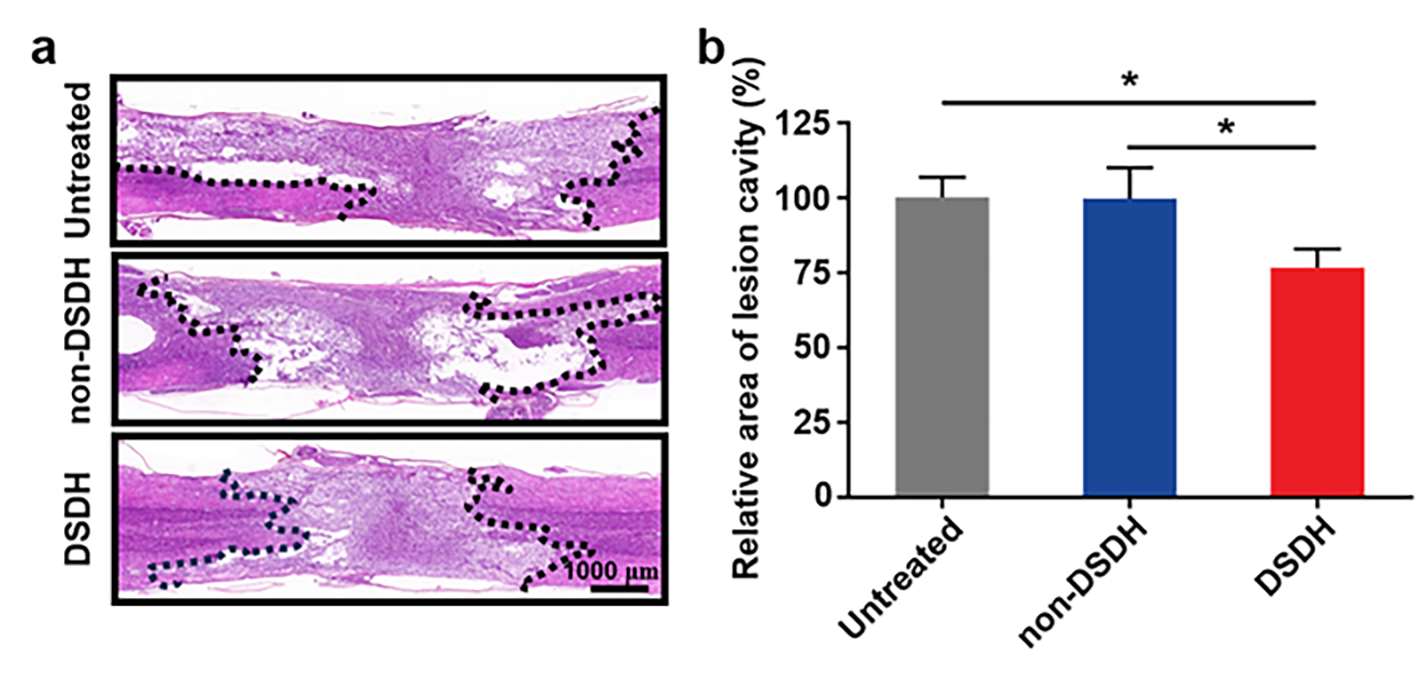


**Figure S13.** (**a**) H&E staining results of spinal cord tissue after 28 days of treatment. (**b**) Percentage of cavity area in the lesion site (n=3). Data are presented as mean ± SD and statistical significance was analyzed via the independent sample t-test; *p<0.05.


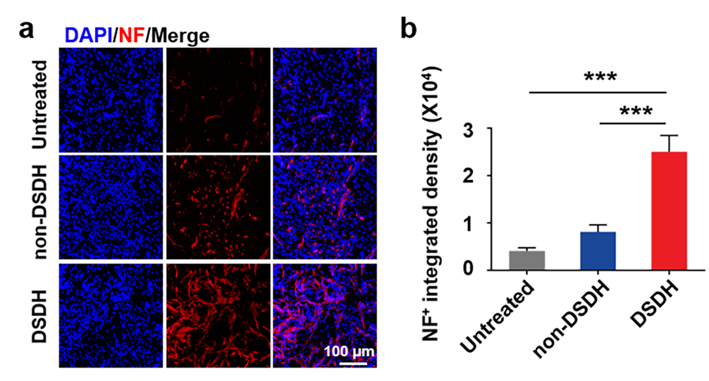


**Figure S14.** Immunofluorescence staining confirms that DSDH promotes neurofilaments regeneration. (a) NF expression in the lesion area. (b) NF^+^ integrated density in the injury site of the untreated, non-DSDH, and DSDH groups (n = 3). Data are presented as mean ± SD and statistical significance was analyzed via one-way ANOVA with Tukey’s multiple comparison test; ***p < 0.001.


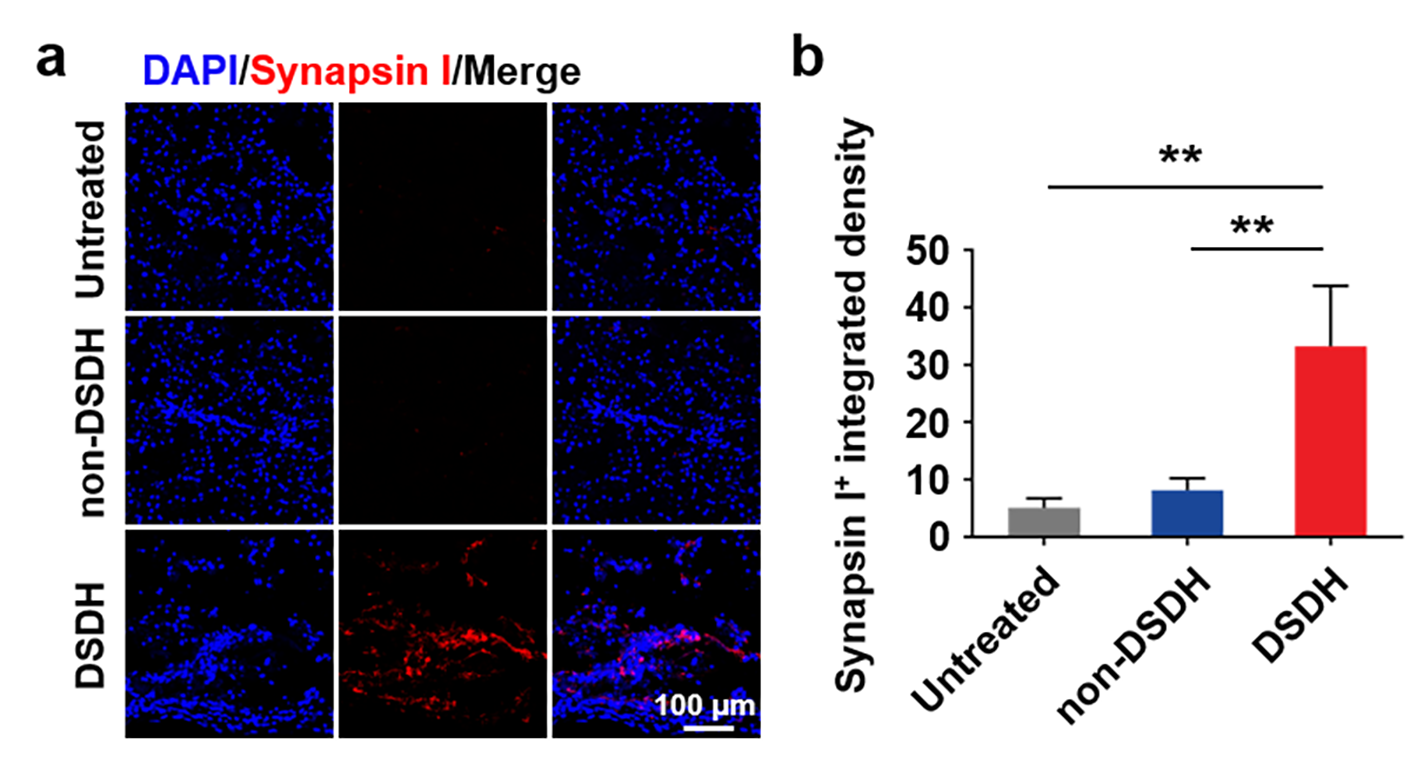


**Figure S15.** Immunofluorescence staining confirms that DSDH promotes synaptic formation. (a) Synapsin I expression in the lesion area. (b) Synapsin I^+^ integrated density in the injury site of the untreated, non-DSDH, and DSDH groups (n = 3). Data are presented as mean ± SD and statistical significance was analyzed via one-way ANOVA with Tukey’s multiple comparison test; **p < 0.01.


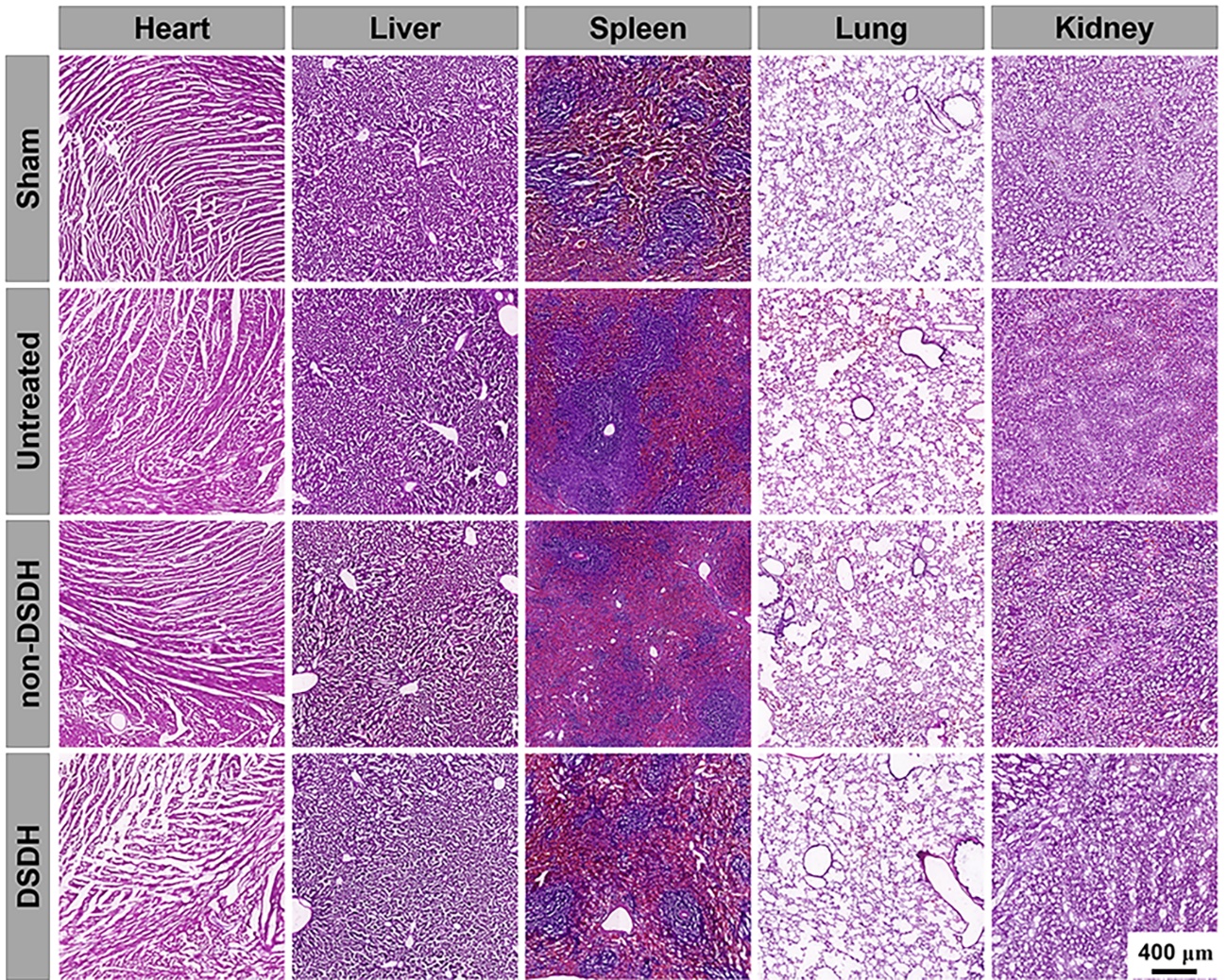


**Figure S16.** H&E staining of heart, liver, spleen, lung, and kidney after 28 days of treatment.

**Table S1.** Physicochemical properties of nanoparticles prepared with different mass ratios of MH to fucoidan.

| **MH:Fuc** | **Size (nm)** | **PDI** | **Zeta (mV)** | **EE (%)** |
| --- | --- | --- | --- | --- |
| 1:2.5 | 245.6±82.9 | 0.426±0.143 | -26.7±1.5 | 24.08% |
| 1:5 | 119.9±1.0 | 0.510±0.045 | -30.9±1.3 | 63.54% |
| 1:10 | 119.4±0.8 | 0.372±0.030 | -30.4±0.3 | 93.18% |
| 1:15 | 121.3±0.1 | 0.214±0.020 | -26.1±0.1 | 95.76% |
| 1:20 | 151.5±5.3 | 0.271±0.019 | -32.0±1.9 | 95.94% |
